# Early cell-autonomous and niche-mediated epithelial response to influenza infection in primary alveolar organoids

**DOI:** 10.1172/jci.insight.201739

**Published:** 2026-07-02

**Authors:** Amber Elitz, Sharlene Fernandes, Kathleen C.S. Cook, Helen I. Warheit-Niemi, Barbara Zhao, Andrea Toth, Amanda L. Zacharias, William J. Zacharias

**Affiliations:** 1 Medical Scientist Training Program,; 2 Development, Stem Cells, and Regenerative Medicine Graduate Training Program,; 3 Perinatal Institute,; 4 Division of Pulmonary Biology,; 5 Department of Pediatrics,; 6 Division of Pulmonary, Critical Care and Sleep Medicine, and; 7 Department of Internal Medicine, University of Cincinnati College of Medicine, Cincinnati, Ohio, USA.

**Keywords:** Infectious disease, Pulmonology, Adult stem cells, Bioinformatics, Influenza

## Abstract

Influenza A virus (IAV) infection is a major cause of morbidity and mortality for patients worldwide. Alveolar type 2 (AT2) cells are the preferential target of IAV as part of the pathogenesis of viral pneumonia and acute respiratory distress syndrome (ARDS). Early IAV infection of alveolar cells has been challenging to model both in vitro and in vivo. To address this challenge, we used a combination of murine and human primary alveolar organoids to define methods for robust IAV infection and evaluated cell-autonomous consequences of IAV using a temporal series of multiome paired single-nucleus RNA and ATAC sequencing assays. Infected AT2 cells demonstrated conserved changes defined by early loss of surfactant secretion, decreased lipid biogenesis, a rapid burst of antiviral response, and late virus-mediated suppression. Surprisingly, uninfected AT2 cells underwent substantial transcriptional and epigenomic changes in IAV-treated cultures, leading to transition to damage-associated cell states within hours via a process driven by the inflammatory milieu of murine organoids. Together, these data provide methods for high-fidelity modeling of IAV infection in alveolar cells and defined a conserved AT2 cell response signature to IAV with implications for ARDS pathogenesis.

## Abstract 2

This work evaluated the gene expression and epigenome changes caused directly by flu virus on lung epithelial cell organoids to identify mechanisms that could improve resilience to influenza pneumonia

## Introduction

Influenza A viruses (IAVs) infect nearly 1 billion people annually, and over 3 million of those infections cause severe lower respiratory tract disease, or viral pneumonia (1, 2). Viral pneumonia leads to disruption of the critical alveolar gas exchange surface via direct infection of alveolar type 2 (AT2) pneumocytes in the distal lung. Many patients with viral pneumonia will develop respiratory failure and require ICU care due to acute respiratory distress syndrome (ARDS) (3). ARDS is characterized clinically by bilateral pulmonary infiltrates and hypoxemia resulting from fluid and immune cell influx into the alveolar space and associated alveolar collapse (atelectasis) (4, 5). Coordinated regeneration is required to restore gas exchange and alveolar structure (6) during recovery. Failure of these regenerative mechanisms is associated with increased risk of secondary bacterial pneumonia (2) and pulmonary fibrotic remodeling leading to fibroproliferative ARDS (7). These complications are associated with high morbidity and mortality.

Previous studies have attributed the extent of injury following IAV infection and risk of subsequent ARDS to many factors (2), including immune-mediated tissue damage, lysis of infected cells impairing the alveolar barrier, loss of vascular integrity, and physiological stress resulting from hospital interventions like mechanical ventilation (4, 5, 7–9). The knowledge that these factors contribute to disease has not yet led to improved anti-influenza therapies. Extensive literature has highlighted that even patients who recover from viral pneumonia may have profound, long-lasting respiratory dysfunction (7, 10, 11). These limitations suggest that new approaches are needed to study IAV disease pathogenesis prior to onset of ARDS, and to guide the development of new therapies.

Pathologically, IAV causes extensive injury to the alveolar epithelium during viral pneumonia. The primary role of the alveolus is to maintain ventilation via the air-facing gas exchange surface (12), a role that requires high surface area alveolar type 1 (AT1) cells to interact with alveolar capillaries to provide functional ventilation. AT2 cells produce surfactant proteins to regulate surface tension in the alveolus, preventing atelectasis (13). These cell populations are maintained through activity of Wnt signaling in AT2 progenitors (14, 15), which can enter the cell cycle, replicate, and replenish AT1 and AT2 cells following IAV injury of the distal lung. After infection, the IAV genome replicates in the nucleus of infected cells. Recent studies have shown that host stress (16) and viral nonstructural protein 1 (NS1) contribute to readthrough error and repression of host transcription in infected cells, leading to changes in cellular chromatin structure and gene expression (17, 18). AT2 cells additionally play a vital role in pulmonary immunity (19). During IAV infection, virally infected cells present IAV-derived antigens at the cell surface, activating innate immune cells in the alveolus and leading to recruitment of circulating immune cells that target and clear infected cells. Both tissue-resident and recruited immune cells secrete cytokines that signal to other cells in the infected alveolus (20). Thus, damage to alveolar epithelial populations, either through direct, cell-autonomous mechanisms or the inflammatory milieu, results in the loss of alveolar gas exchange, regenerative function, or surfactant secretion. These cellular processes underlie key aspects of the pathogenesis of ARDS (21). However, the direct alveolar epithelium–specific dynamics of IAV infection are challenging to study in vivo due to immune-mediated injury and clearance of infected cells. Conversely, existing 2-dimensional in vitro systems do not model the cellular relationships of the alveolar epithelium, can require viral activation to improve infectivity of cells (22, 23), and poorly model AT2 cell state and function.

To address these challenges, we present here findings describing optimization and modeling of IAV infection in primary murine and human alveolar epithelial organoids (AEOs). Using this in vitro infection platform, we characterized both cell-autonomous and paracrine effects on cell state, transcriptome, and epigenome using multiomic single-nucleus RNA sequencing (snRNA-seq) and assay for transposase-accessible chromatin using sequencing (snATAC-seq). We find that IAV-infected AT2 cells differ transcriptionally and epigenomically from uninfected AT2 cells, and that many of these changes are driven by inflammatory signaling occurring within 24 hours of IAV exposure in vitro. Using paired analysis of RNA expression and chromatin accessibility data, we define a conserved AT2 response to infection associated with changes in surfactant production and immune-mediated antiviral responses, delineating key IAV-mediated mechanisms underlying ARDS pathology and providing a large, integrated multiomic dataset of IAV-mediated cellular responses for exploration and community use.

## Results

### Optimizing influenza infection in complex AEOs.

**Figure 1:**
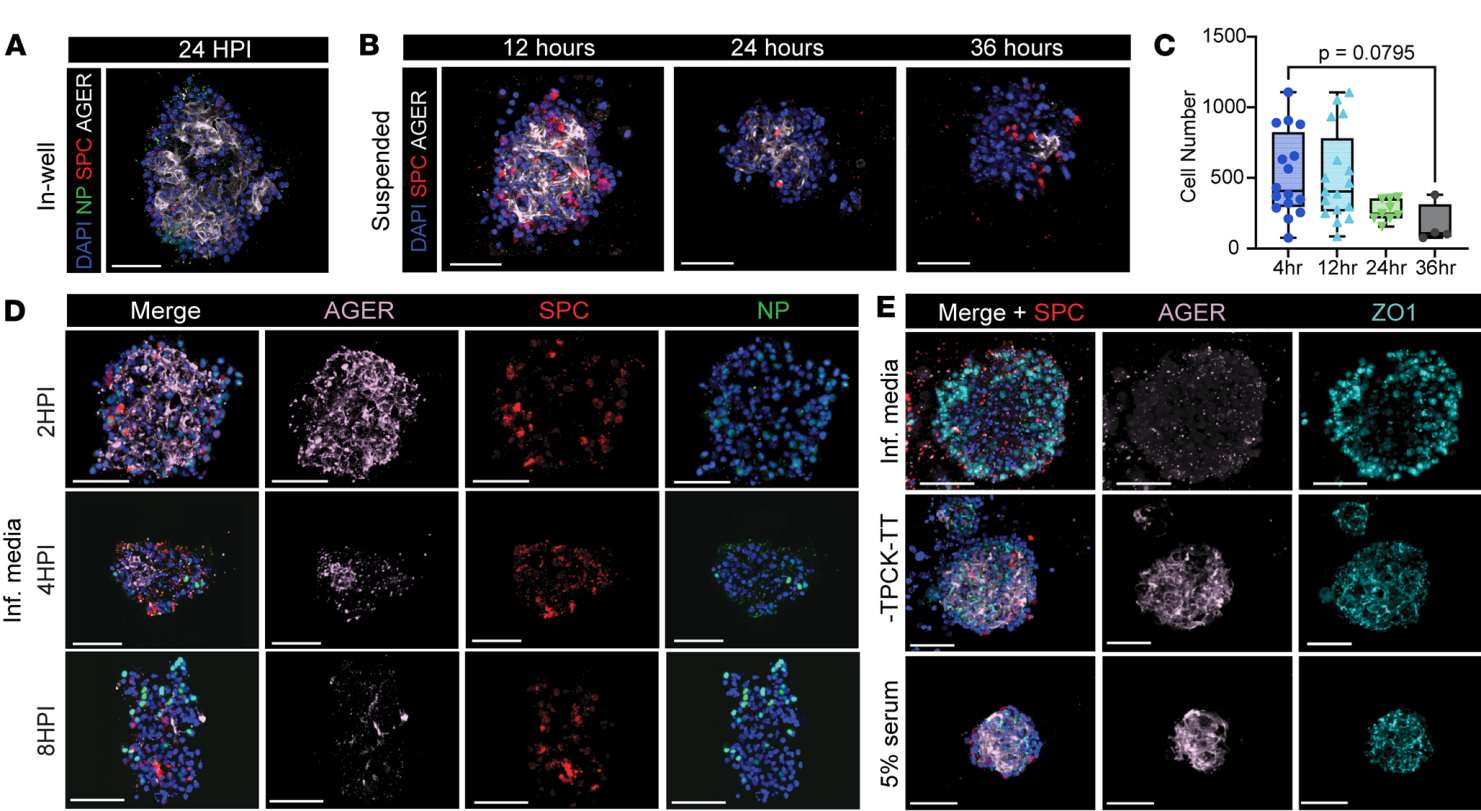
Optimization of influenza A (IAV) infection conditions for alveolar organoids (AEOs). (**A**) Treatment of AEOs with IAV in media placed on apical surface of Transwell above Matrigel plug. Minimal NP expression is evident at 24 hpi. (**B**) Morphology of AEOs in suspension culture after liberation from Matrigel. AT1 and AT2 cell morphology is maintained for 24 hours after suspension but has decreased by 36 hours. (**C**) Cell number per organoid in suspension culture. We noted a substantial loss of cell number by 36 hours after liberation. (**D**) Cell state of AEOs with infection of IAV using SAGM base with TPCK-treated trypsin and lacking serum (Inf. media). Suspension in this medium allowed flu infection by 8 hours (NP) but led to loss of AT1 cells (AGER) without loss of AT2 cells (SPC). (**E**) Impact of different media compositions on cell junctions in AEOs. Infection media (top row) led to loss of AGER and associated loss of ZO-1–marked cell surface tight junctions. Removal of TPCK-treated trypsin or addition of 5% serum prevented these changes. Scale bars: 50 μm. SPC, surfactant protein C (AT2 marker); AGER, advance glycosylation end-product–specific receptor (AT1 marker); NP, IAV nucleoprotein (infected cell marker); ZO-1, zonula occludens-1 (cell surface tight junction marker).

To model IAV infection in AEOs, we generated AEOs by sorting primary AT2 progenitor cells from adult mice and combining with expanded primary mesenchyme in Matrigel and small airway epithelial cell growth medium (SAGM), as previously described (24). After culturing AEOs for 21–28 days, we attempted infection in AEOs still within the Matrigel plug by adding H1N1 PR8 IAV at a multiplicity of infection (MOI) of 10 (1.025 × 10^6^ PFU) to the top of each well in spiked SAGM (see Methods). We observed that few cells were infected and expressing IAV nucleoprotein (NP) 24 hours post infection (hpi) (Figure 1A), suggesting that viral diffusion through the Matrigel was too inefficient for robust AEO cell infection. This has been overcome in previous studies by using cultures with thin layers of matrix (25, 26) or by applying the infection to organoids or cells in suspension (27–29). To improve infection kinetics in our AEOs, we removed the mature ones from the matrix and transitioned the isolated AEOs to suspension culture in SAGM. We evaluated protein expression of AGER and surfactant protein C (SPC) to detect AT1 and AT2 cells, respectively. Transition to suspension minimally disrupted AEO cell number and cavity integrity within the first 24 hours, with a general loss of AT1 cells and a substantial loss of cell number per AEO by 36 hours (Figure 1, B and C). We therefore chose to restrict our evaluation to the first 24 hours of suspension culture. This had the advantage of focusing our analysis on early impact of IAV.

We next sought to optimize our AEO infection media conditions. Published protocols in monolayer culture and lung spheroids have demonstrated that L-1-tosylamide-2-phenylethyl chloromethyl ketone–treated (TPCK-treated) trypsin cleaves influenza entry factor HA0 to expose the viral particle binding sites and facilitates infection initiation in cell lines lacking key transmembrane proteases (30). The addition of serum to infection media for IAV has been suggested to impede viral infection, likely by quenching trypsin activity (30). We therefore evaluated an “infection media” that was both serum-free and contained TPCK-treated trypsin. We found that AEOs, particularly the AT1 population, were extremely sensitive to disturbances in mechanical tension induced by trypsin. While the AEOs retain AT1 structure and specification in suspension (Figure 1B), the addition of TPCK-treated trypsin cleaved cell-cell junctions and produced a selective loss of AT1 cells followed by general organoid degradation within 8 hours (Figure 1D). Removal of trypsin from the media or addition of 5% serum for neutralization protected AT1 morphology in AEOs (Figure 1E). We therefore decided to proceed with infection modeling in suspension culture using complete SAGM containing serum.

### Infection dynamics and cellular specificity of IAV in vitro in AEOs.

**Figure 2:**
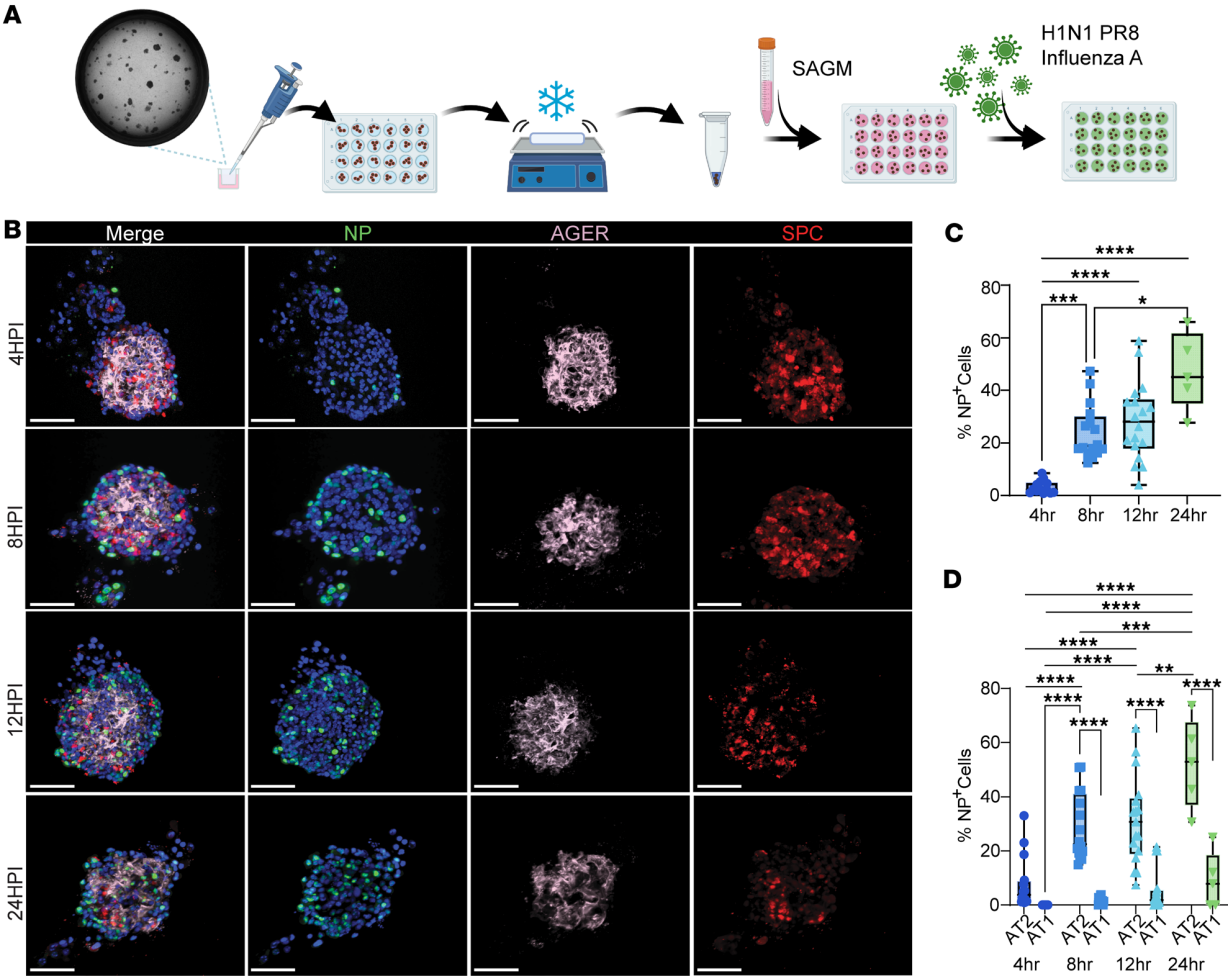
Temporal dynamics and cellular specificity of IAV infection in AEOs. (**A**) Schematic of method for liberation and treatment of AEOs with IAV. (**B**) AEOs treated with H1N1 develop progressively increased influenza infection as measured by nuclear expression of H1N1 NP across 24 hpi. (**C**) Quantification of NP^+^ nuclei at each time point of infection. (**D**) Cell-type-specific infection in AEOs. More AT2 cells are infected than AT1 by 8 hpi and thereafter. Scale bars: 50 μm. SPC, surfactant protein C (AT2 marker); AGER, advance glycosylation end-product–specific receptor (AT1 marker); NP, IAV nucleoprotein (infected cell marker); SNA, *Sambuccus* *nigra* agglutinin (flu binding motif α-2,6-sialic acid). *P < 0.05; **P < 0.01; **P < 0.001; ****P < 0.0001 by ANOVA with prespecified multiple comparisons; 1-way ANOVA (**C**); 2-way ANOVA (**D**).

Using our optimized infection conditions, we performed an infection time series in AEOs in SAGM suspension (Figure 2A) at 4, 8, 12, and 24 hpi, and followed infection kinetics by whole-mount IHC to detect (Figure 2B) and quantify (Figure 2, C and D) NP-positive nuclei. We reasoned that by 24 hpi, host cells have undergone a full viral life cycle, with the majority of infected cells experiencing a period of continuous viral replication (31), suggesting that key changes in early IAV pathogenesis would likely be captured by this time point. The percentage of NP^+^ cells per AEO progressed from less than 10% at 4 hpi to 24% at 8 hpi, 29% at 12 hpi, and more than 40% at 24 hpi (Figure 2C). These results suggest that cells expanded in primary AEO culture retained sufficient expression of key transmembrane proteases that perform the HA cleavage to enable viral binding in the absence of TPCK-treated trypsin. Furthermore, these kinetics indicate sufficient rates of infection for downstream analysis, without the additional stress on the cells of re-embedding in matrix for a longer incubation. This is a critical distinction from some previously published infection models in which organoid shearing/breaking was optimal for organoid infectivity (32–34). IHC demonstrated infection primarily in AT2 cells when corrected for population size (Figure 2D). To assess viral binding availability, we performed staining with the lectin *Sambucus* *nigra* agglutinin (SNA) to evaluate the presence of the primary IAV binding moiety, α-2,6-sialic acid (Supplemental Figure 1A; supplemental material available online with this article; https://doi.org/10.1172/jci.insight.201739DS1). AT2 cells demonstrated increased SNA labeling compared with AT1 in AEOs. This distribution was maintained following a 24-hour suspension with no noteworthy changes to α-2,6-sialic acid polarity on the AT2s (Supplemental Figure 1B). To assess α-2,6-sialic acid expression in AT2 and AT1 cells in vivo, we performed flow cytometry from freshly isolated epithelium from wild-type mice (Supplemental Figure 2, A and B). To confirm this specificity in cell infectivity in vivo, infected R26R-EYFP mice with a PR8 strain harboring Cre and evaluated YFP expression per cell type by flow cytometry (Supplemental Figure 2A). We confirmed more expression of SNA in AT2 cells in vivo (Supplemental Figure 2C) and found more infected AT2 cells than AT1 at 72 hpi in vivo (Supplemental Figure 2D). Taken together, these data demonstrated a clear progression of IAV infection in AEOs across the 24 hours in suspension, which replicated the cellular targeting of IAV in the lung in vivo.

### Defining trajectories of cellular injury in IAV AEOs with multiome sequencing.

To evaluate changes in gene expression and chromatin accessibility in infected AEOs, we performed multiomic snRNA-seq and snATAC-seq on nuclei isolated from AEOs suspended in SAGM with or without H1N1 at 8, 16, and 24 hpi. The multiome assay generated linked RNA and ATAC data at per-nucleus resolution, allowing us to assess both gene transcript expression and chromatin accessibility per cell. To identify infected cells, we generated a custom mm10 (35, 36) genome alignment file that included the PR8 viral genome to allow detection of cells with active viral replication occurring in the nucleus. After filtering to remove doublets (37) and ambient RNA (38), we recovered 33,926 single-nucleus transcriptomes with a range of 3,642 to 8,793 cells per time point and condition, with a median of 943 unique molecular identifiers (UMIs) (interquartile range 614–1,712) per cell, and a median of 734 genes (interquartile range 509–1,186) per cell. These snRNA-seq depths were below those previously observed with single-cell RNA-seq (scRNA-seq) in AEOs (24) or in vivo after flu infection (21, 39), but provided resolution to define infected cells and simultaneously compare transcriptome and epigenome state. We focused our analysis on major cell states defined by nearest neighbor analysis using both snRNA-seq and snATAC-seq assays.

**Figure 3:**
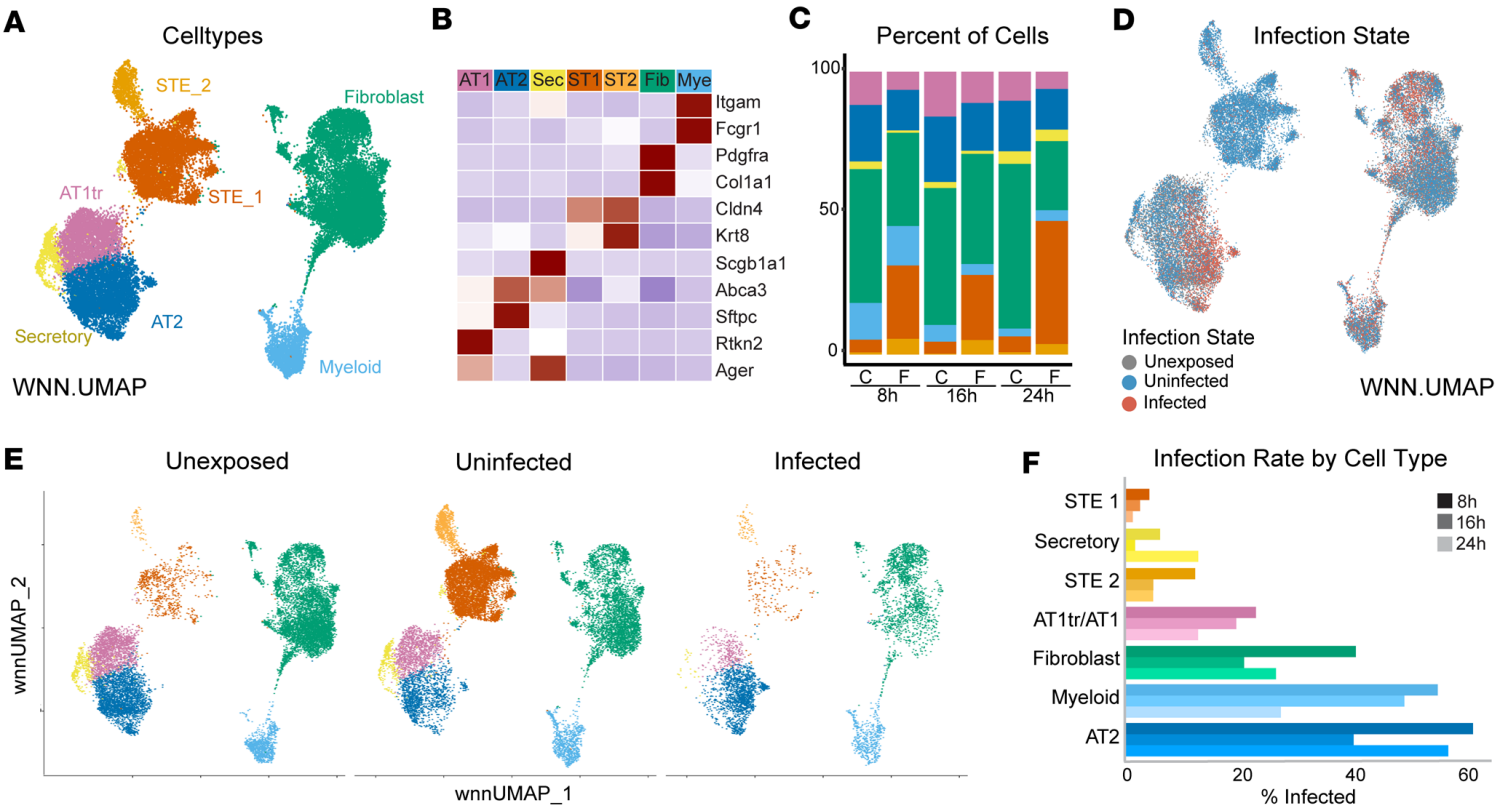
Single-nucleus analysis of flu infection in AEOs. (**A**) UMAP of cells from unexposed and flu-treated AEOs. (**B**) Cell state markers defining cell states shown in **A**. (**C**) Frequency of cell states observed in unexposed (“C”) and flu-treated (“F”) AEOs. (**D**–**F**) Infection states. (**D**) Overall UMAP. (**E**) Individual condition UMAPs showing unexposed AEOs, uninfected cells in flu-treated AEOs, and infected cells in flu-treated AEOs. (**F**) Overall rate of infection per cell type. AT1tr, AT1-like/transitional cells; STE, stressed transitional epithelial cell. Other abbreviations per main text.

Following unbiased clustering (Figure 3A), we identified AT2 cells, as well as a population of AT1 and AT1-like (AT1tr) cells within the epithelium, as expected based on our IHC results (Figure 3B). We also detected the anticipated mesenchymal/feeder cell population, including mouse lung fibroblasts and some myeloid cells that persist in the feeder culture but do not alter infection kinetics (Supplemental Figure 3, A–C). These cells were identified by marker gene analysis as most similar to interstitial macrophages (40). Unique to the IAV-exposed condition, we noted the presence of 2 populations we denoted as stressed transitional epithelial cells (STEs). These populations share a gene expression signature typical of previously published (41) Krt8^High^ (also known as PATS [ref. 42], DATP [ref. 43], or ADI [ref. 44]) cell populations known to arise in diverse forms of lung epithelial cell injury. The presence of these cell states has been described within several days of injury in vivo, but they arise within hours following IAV infection in our AEO system. Furthermore, we detected a distal secretory-like population defined by joint expression of Sftpc and Scgb1a1, most prevalent at the 24-hour time point in unexposed AEOs (Figure 3, A and B). This population appears to arise with acquisition of Scgb1a1 expression in AT2 cells during the unexposed suspension, potentially as a response to suspension culture in the absence of other stressors (Figure 3, B and C). We note that there is likely a higher degree of heterogeneity of cell states present within both the epithelial and fibroblast populations, but the depth of multiome snRNA-seq was insufficient for confident definition of these more refined cell states.

Next, we identified the IAV infection state in cells. We generated an IAV expression score based on the number of IAV genes and expression levels to distinguish 3 classes of cells (see Methods): (a) “Unexposed” cells from IAV-naive AEO cultures, (b) “Infected” cells, which had high levels of flu-specific gene expression, and (c) “Uninfected” cells, which were exposed cells in IAV-treated cultures but did not meet the IAV gene expression thresholds for infection (Figure 3, D and E). Concordant with our AEO and in vivo IHC data, the majority of infected epithelium were AT2 cells with few infected AT1 cells. Surprisingly, we noted the presence of substantial populations of infected fibroblasts and myeloid cells (Figure 3E) with a highest enrichment at the 8-hour time point (Figure 3F). We suspect that these “off-target” infections are due to prolonged saturation with viral particles with no protective extracellular matrix. HA cleavage by epithelial cells may also improve viral availability for infection of mesenchymal lineages. Notably, both STE populations had a very low rate of flu infection.

### Gene expression profiles of infected cells within AEOs.

**Figure 4:**
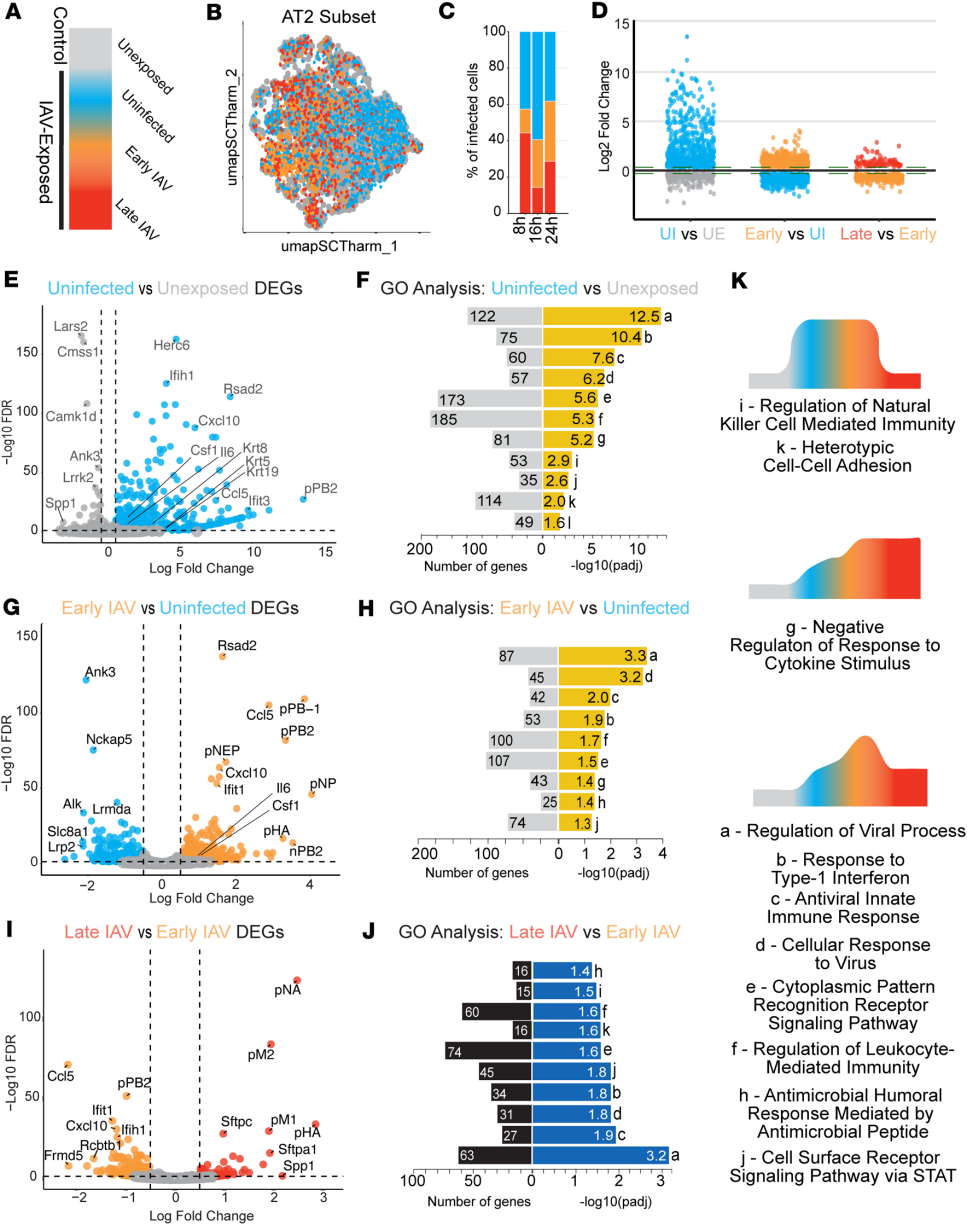
Trajectory of gene expression during IAV infection in AEOs. (**A**) Schematic color scheme of cell states in AEOs. Unexposed, gray; Uninfected, blue; Early IAV, orange; Late IAV, red. (**B** and **C**) UMAP (**B**) and frequency (**C**) showing infection states of AT2 cell subset of AEOs. (**D**) Strip plots showing number of differentially expressed genes (DEGs) and degree of enrichment across AT2 cell states. (**E** and **F**) Volcano plot of DEGs (**E**) and GO Biological Process enrichment (**F**) of genes in Unexposed (gray) versus Uninfected (blue) AT2 cells. (**G** and **H**) Volcano plot of DEGs (**G**) and GO Biological Process enrichment (**H**) of genes Uninfected (blue) versus Early Infected (orange) AT2 cells. (**I**–**J**) Volcano plot of DEGs (**I**) and GO Biological Process enrichment (**J**) of genes in Early Infected (orange) versus Late Infected (red) AT2 cells. Upregulated processes are shown in yellow, and downregulated processes in blue. (**K**) State dynamics of enrichment of GO terms from **F**, **H**, and **J**. Letter to left of process name matches letters to right of bars. Some terms are enriched at multiple time points. Diagrams in **K** show dynamics of change of associated terms (boxes) across cell states in AEOs.

We next turned our attention to defining the pathways associated with infection state in AT2 cells. Within the “Infected” population, we defined cells as “Early” infection based on early gene signatures (expressing PB1 and PB2, PA, and PA-X, NP, NS1, and NEP) for genes involved in poly-A cap snatching, host machinery hijacking and disruption, and nuclear transport. We scored cells as “Late” infection due to expression of later-stage genes (M1 and M2, HA, NA, and PB-1-F2) involved in building the viral particle membrane and membrane proteins in addition to the “early” genes (Figure 4, A–C) (2). The resulting patterns of infection stage at each time point aligned with timelines previously reported in monolayer cell culture models (31). In AT2 cells, we detected “Early Infected” cells at each time point. We interpreted these results to indicate that cells experience ongoing infection while in suspension in IAV-containing media. We detected “Late Infected” cells by 8 hpi, less at 16 hpi, and then again increased at 24 hpi; these data suggest that our system allowed up to 2 full cycles of viral replication by 24 hpi (Figure 4, B and C). 

To define the cell-state-specific effects of IAV infection on AT2 cells, we created pseudobulk profiles of cells scored as Unexposed, Uninfected, Early IAV, or Late IAV infection, ensuring even contribution of cells from each time point combined into the artificial replicates to prevent time point biases. We identified differentially expressed genes (DEGs) that we defined by an adjusted *P* value of less than 0.05 and a fold change (FC) of greater than 1.25 (Figure 4D) within these populations. Surprisingly, we noted the most DEGs comparing the Unexposed to Uninfected cells, with fewer changes between Uninfected to Early Infection, and few genes changed between Early and Late infection.

To evaluate major pathways associated with genes regulated across the course of AT2 infection, we performed Gene Ontology (GO) Biological Process (BP) term enrichment analysis (45, 46). Uninfected AT2 cells upregulated genes associated with pathways of AT2 stress response and immune signaling, including viral and interferon response (Figure 4, C and D). These terms were driven by leading-edge genes, including Krt8, Krt19, Cxcl10 (IP-10), and Ccl5 (RANTES) (Figure 4, E and F). Many of these genes and pathways were further upregulated in Early IAV cells compared with Uninfected AT2 (Figure 4, G and H), but then downregulated in Late IAV (Figure 4, I and J). The Late infection condition was instead defined by genes associated with pathways controlling protein folding, with higher expression of several heat shock protein genes and ER stress markers (Figure 4, I and K). Finally, we noted a substantial initial increase in intracellular markers of type 1 interferon response in AT2, including Irf5 and Isg15. Consistent with published results, this signature is then downregulated in late infection, which has been reported as a consequence of virus-mediated suppression (47). Taken together, these data unite several proposed mechanisms underlying influenza response in AT2 cells (47–50) (Figure 4K).

### Cell epigenomic state changes in IAV infection.

**Figure 5:**
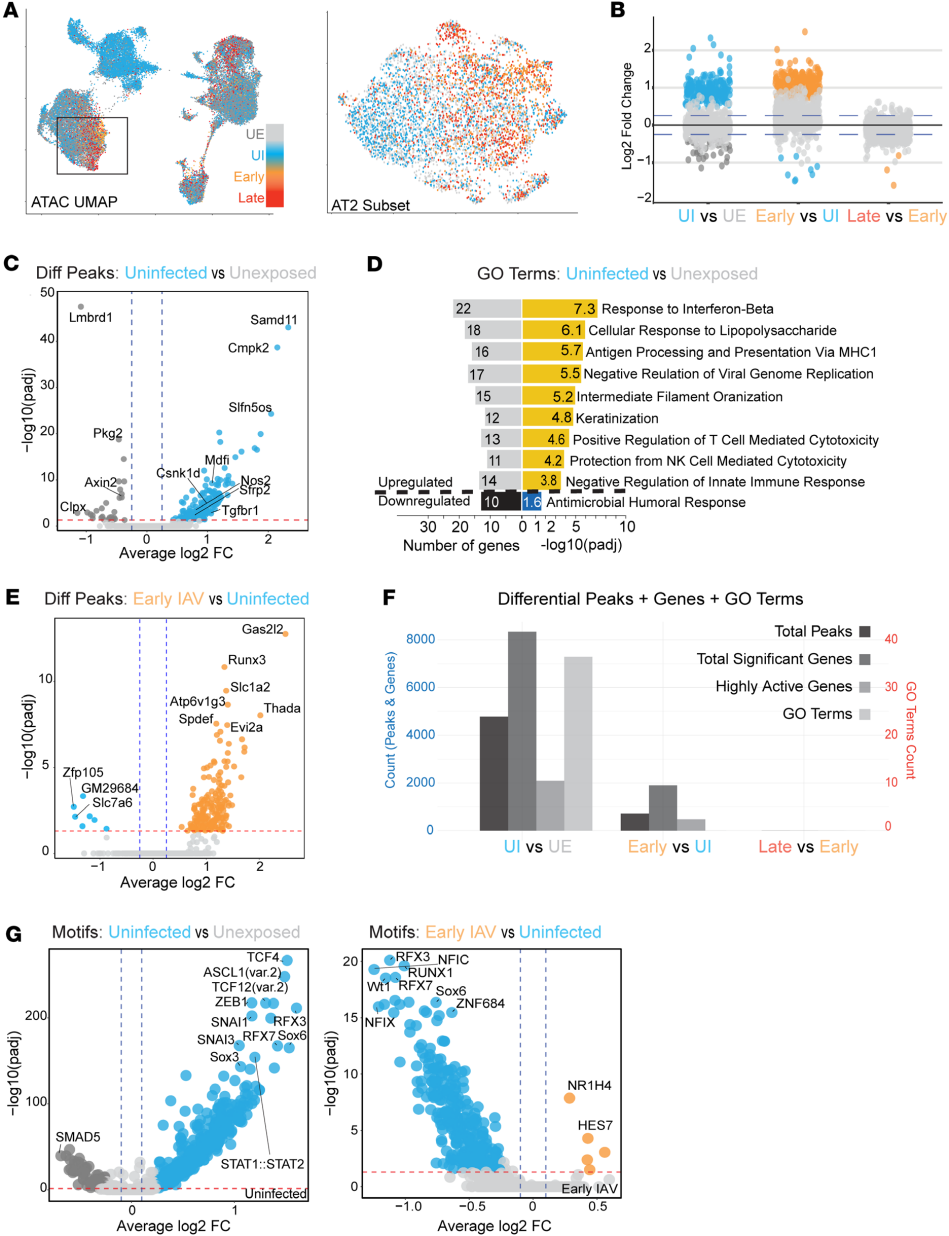
Dynamics of chromatin changes during IAV infection in AEOs. (**A**) Overall UMAP and AT2-only UMAP based on ATAC assay showing cell infection state. (**B**) Strip plot showing number and significance of differential peaks across AT2 cell states. (**C** and **D**) Volcano plot (**C**) and GO terms (**D**) showing nearest neighbor genes to differentially open chromatin enriched in Uninfected compared to Unexposed AT2 cells. (**E**) Volcano plot of nearest neighbor genes to differentially open chromatin in Early infected compared to Uninfected AT2 cells. (**F**) Bar chart showing the number of differentially open peaks, nearest neighbor genes, top 25% active (highly active) genes, and associated enriched GO terms. No enriched GO terms are associated with newly open chromatin in IAV-infected AT2 cells, and no differentially open chromatin regions passed thresholding for Early versus Late infection. (**G**) Motifs enriched in differentially open chromatin in Uninfected versus Unexposed (left) or Early infected versus Uninfected (right) AT2 cells.

We next turned our attention to the epigenetic consequences of IAV infection by comparing Unexposed, Uninfected, and Infected AT2 cells in snATAC-seq data (Figure 5A). We identified differentially open regions of chromatin with a log_2_FC of greater than 0.25 and an FDR threshold of 0.05 within pseudobulk chromatin signatures from each of these cell states. Using an ArchR-like (49) approach to identify the top 25% most active (highly active) differentially regulated peaks (Figure 5B), we observed the largest difference in open chromatin between Uninfected versus Unexposed AT2 (Figure 5C). Taken with the snRNA-seq findings, these data implied that a majority of chromatin regulation was related to detection and early response to IAV in the system rather than direct infection in AT2 cells. We found marked changes in chromatin near MHC- and IRF-related genes in Uninfected cells. These genes are important for AT2 immune function and the associated RNAs were upregulated in Early Infected cells (Figure 4G and Figure 5C). GO terms associated with these differential peaks included terms associated with response to IL-1β, TNF-α, and suppression of viral genome replication. Chromatin in Uninfected AT2s was also more open near genes associated with keratinization, intermediate filament process, cellular response to inflammatory stimuli, and antigen presentation concordant with the observed changes in gene expression by snRNA-seq (Figure 4C and Figure 5D).

We next turned our attention to chromatin changes induced by flu infection, comparing Uninfected and Early infected cells. We noted a large number of differentially accessible peaks in Early IAV (Figure 5E), but noted that these newly accessible peaks were not associated with enrichment of any specific GO BP terms (Figure 5F). We noted that while some chromatin regions were differentially accessible in Early IAV compared with Uninfected, none of these peaks passed our stringent thresholds for significance (Figure 5F). Recent reports have demonstrated that IAV inhibits transcriptional termination in cell lines, leading to widespread readthrough transcription and marked chromatin accessibility changes (17, 18). While we can not directly assess readthrough transcription in our snRNA-seq data, the randomly distributed chromatin changes present in IAV-infected cells likely represent the sequelae of readthrough transcription in infected AT2 cells.

Motif analysis of Unexposed compared to Uninfected AT2s predicted increased activity of transcription factor classes associated with cell shape change (Zeb1) (51) and MHC class II expression (Rfx3/7) (52), concordant with the anticipated need for injury response (Figure 5G). These data suggested that following viral detection in the milieu, AT2 cells are primed via inflammatory signaling to increase chromatin accessibility in key immune-activating factors that increase in transcription prior to and immediately following infection. Consistent with the transcriptome and peak accessibility data, motif analysis in Early IAV demonstrated very few enriched binding motifs within these differentially open regions (Figure 5G).

### Stressed transitional cells arise from uninfected AT2 during response to IAV in AEOs.

As noted above, we observed a large population of STEs exclusively in IAV-exposed AEOs. The STE state has been extensively described in recent pulmonary regeneration literature, where STEs have been shown to both participate in productive regeneration as well as accumulate in more chronic injury (41–44, 53–55). STEs arise within days in vivo, but were present by 8 hpi in AEOs, providing an opportunity to evaluate signatures and possible origins of STEs in IAV-infected AEOs.

**Figure 6:**
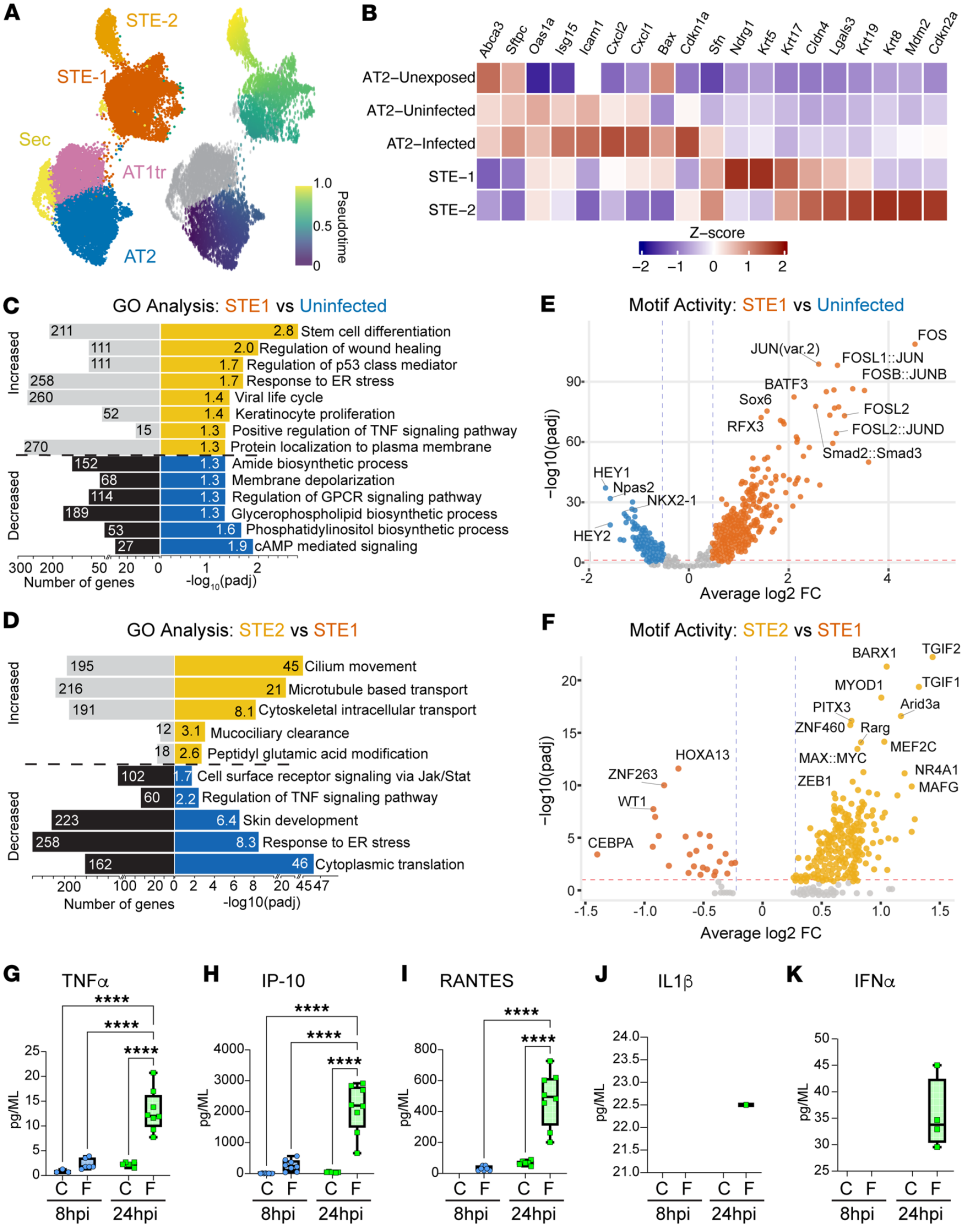
STEs arise from uninfected AT2 cells driven by inflammatory signaling. (**A**) Epithelial-only UMAP (left) and pseudotime trajectory (right) suggesting that STEs arise from AT2 cells in IAV-treated AEOs. (**B**) Marker gene expression of a series of AT2 and STE markers defining cell state in AEOs. (**C** and **D**) GO Biological Process terms based on DEGs for (**C**) Uninfected AT2 cells versus STE1s and (**D**) STE1s versus STE2s. (**E** and **F**) Motif activity enrichment based on differentially open chromatin (**E**) in Uninfected AT2s versus STE1s and (**F**) and motif activity enrichment based on differentially open chromatin (**F**) in STE2s versus STE1s. Major reported regulators of STE transition, including reduction in NKX2-1 activity, IL-1β signaling driving FOS/JUN signaling, and TGF-β signaling are identified in STE1s versus AT2 cells. (**G**–**K**) Cytokine ELISAs demonstrating enrichment of multiple IAV-associated cytokines in the media of IAV-infected AEOs. Compare to Supplemental Figure 5. ****P < 0.0001 by 2-way ANOVA with prespecified multiple comparisons.

Both STE populations were predicted by pseudotime analysis of snRNA-seq data to arise from the AT2 population (Figure 6A). Although our system does not include a lineage tracing marker to validate this predicted relationship, uninfected AT2s showed enrichment at the ATAC level for gene programs associated with the transition to the STE state, including keratinization and intermediate filament–associated gene categories relative to unexposed AT2s (Figure 5, C and D). STE populations in AEOs were defined by RNA expression of cytokeratins 5, 8, and 19 among other established epithelial stress markers (Figure 6B). Both populations expressed components of the “typical” DATP/PATS/ADI/ABI cell (41–44, 53–55) profile described in the literature; however, the STE2 population was particularly Krt8^hi^ and marked by expression of Cdkn1a, Cdkn2a, and Sfn, markers that have been reported in more pathogenic STEs. Both STE populations expressed low levels of the recovery markers Isg15 and Oas1a (Figure 6B), similar to uninfected AT2 cells, suggesting STEs do not arise from AT2 cells recovered from IAV infection.

When we compared DEGs in STEs compared to uninfected AT2 cells (Figure 6C), we noted enrichment of genes associated with ER stress response pathways, inflammatory signaling, pathways associated with organization of keratins, and p53 signaling. All of these signaling pathways have been associated with STE acquisition in previous literature, supporting the model that STEs arise from uninfected AT2s. We noted downregulation of AT2-associated pathways, including lipid metabolism, secretory regulation, and ion transport as well as terms associated with key aspects of ARDS pathogenesis, including local surfactant deficiency and alveolar edema. The shift to STE2 from STE1 was primarily characterized by cytoskeletal, microtubule, and ciliary terms, and a reduction in niche signaling response in the TNF and JAK/STAT pathways (Figure 6D).

### An inflammatory signaling milieu drives STE acquisition in IAV-infected AEO.

We next assessed differential motifs found in chromatin open in STE1 relative to uninfected AT2 cells to predict transcription factor activity (Figure 6E). Motif analysis demonstrated a reduction in predicted NKX2-1 activity and increases in predicted regulation by FOS:JUN and SMAD2:SMAD3 motifs in STE1. STE acquisition has been reported following TGF-β signaling upstream of SMAD2/3 (41), and after loss of function of NKX2-1 (24), suggesting similarities in the gene programs driving STE acquisition following IAV infection in AEOs and in other injury models. Interestingly, STE2-enriched motifs included high predicted activity for TGF-β target genes and feedback inhibitors TGIF1 and TGIF2 (Figure 6F), suggesting modulation of TGF-β signaling may underlie some differences separating the STE1 versus STE2 states. RNA expression of Tgfb1 was not changed in snRNA-seq data and activated TGF-β ligand was not changed in stroma exposed to IAV based on ELISA (Supplemental Figure 4), suggesting that changes in TGF-β processing or response might be responsible for these changes.

Although the specific trigger for STE acquisition in lung injury is unknown, multiple studies have suggested an inflammation-driven process. Previous literature has directly demonstrated a role for IL-1β (43) signaling in STE transition. FOS/JUN activation is a hallmark of cellular response to IL-1β and other proinflammatory cytokines, leading to the hypothesis that inflammatory cytokines induced by influenza may participate in AT2 to STE transitions. To determine which proinflammatory factors are present in the milieu of infected AEOs, we compared RNA expression (Supplemental Figure 5) to protein abundance by ELISA (Figure 6, G–K, and Supplemental Figure 5) for a panel of inflammatory cytokines in IAV-treated or untreated cultures. Protein expression of IFN-α and IL-1β was only detectable in IAV-treated cultures at 24 hpi, and RANTES, TNF-α, and IP-10 were detectable at multiple time points but significantly upregulated only at 24 hpi (Figure 6, G–K). We observed upregulation of RNA for multiple proinflammatory cytokines in fibroblasts in IAV-infected cultures, including the Type 1 interferons IFN-α and IFN-β, IL-6, IP-10/CXCL10, and RANTES/CCL5. We also noted increased RNA expression for several overlapping and additional cytokines from myeloid cells in AEOs, including IL-1β, TNF-α, RANTES, and MIP1α and MIP1β. These data support the conclusion that the AT2-STE transition in IAV-exposed AEOs was likely driven by an inflammatory signaling milieu.

### Generation and characterization of IAV infection in primary human AEOs.

**Figure 7:**
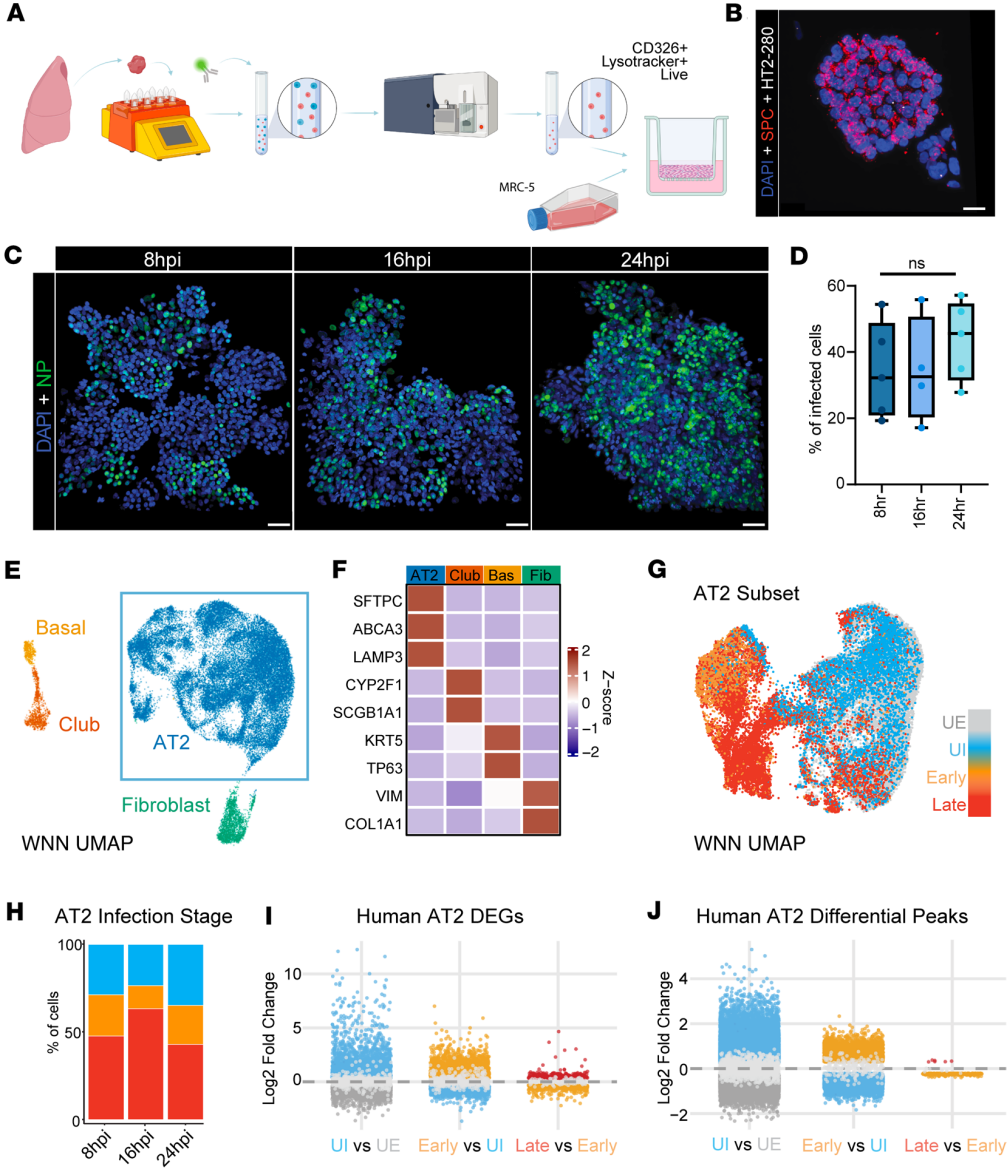
Single-nucleus analysis of flu infection in human AEOs. (**A**) Schematic describing the coculture of freshly sorted human epithelial cells with MRC5 fibroblasts from a single 4-year-old donor for creation of hundreds of human AEOs. (**B**) IHC demonstrating clear punctate SPC expression in human AEOs; we noted relatively low-level HT2-280 expression in established AEO culture. (**C** and **D**) Dynamics of PR8 IAV infection in AEOs, showing high levels of nuclear NP expression (**C**), which is quantified in **D**. (**E** and **F**) UMAP projection (**E**) and cell marker gene expression (**F**) of cell states found in human AEOs. (**G** and **H**) UMAP of AT2 cell subset (**G**) and quantification (**H**) showing imputed flu infection state as was done for murine organoids. (**I** and **J**) Strip plots showing differentially expressed genes (**I**) or differentially accessible chromatin regions (**J**) in Uninfected versus Unexposed, Early Infected versus Uninfected, and Late Infected versus Early Infected human AT2 cells. Scale bars: 50 μm. Statistics in **D** via 1-way ANOVA with prespecified multiple comparisons.

Next, we used published methods (15, 56, 57) to generate primary AEOs from a single healthy 4-year-old human lung that was not utilized for clinical transplantation. We isolated human AT2 cells via FACS (EpCAM^+^, LysoTracker^+^, Live cells) from otherwise healthy lung (Figure 7A, see Methods for donor details). Isolated AT2 cells were cultured in similar conditions to murine cells and using MRC5 cells as mesenchymal feeder cells. These human cultures grew faster than the murine-derived AEOs and generated organoids comprised of numerous AT2 cells (Figure 7B) without the AT1 differentiation or internal complexity or cavitation observed in mouse AEO cultures. We then proceeded to IAV infection in these human AEOs.

To control for virus-specific factors, we used the same PR8 influenza used for murine AEO infection. PR8 is adapted from human pandemic H1N1 but is a mouse attenuated virus and our human AEOs achieved more than double our murine average colony-forming efficiency, creating dense cultures. We therefore doubled the viral load to 2.05 × 10^6^ PFU/well to ensure a similar MOI in human AEOs to that in murine AEOs. Human AEOs underwent efficient infection, with more infected AT2 cells present at 8 hpi and 16 hpi compared with mouse AEOs, but a peak at 24 hpi similar to mouse (Figure 7, C and D). We then proceeded to sequencing via multiome paired snRNA-seq and snATAC-seq from pools of over 300 individual AEOs per condition.

Following nuclei isolation using the same protocol as for mouse AEOs, our snRNA-seq data contained 38,610 single-cell transcriptomes with a range of 5,793 to 7,478 cells per time point and condition, with a median of 3,527 UMIs (interquartile range 1,749–4,795) per cell, and a median of 1,927 genes (interquartile range 1,134–2,374) per cell. Over 75% of cells collected were identified as AT2s with the expected fibroblast presence from the MRC5 coculture (Figure 7, E and F). We observed some airway cell contamination of basal and secretory cells, which were morphologically apparent in human AEO wells and present in both control and IAV-treated cultures, suggesting they originated in the culture prior to the onset of IAV infection. Notably, we did not observe the development of an STE population in the human AEO culture. We hypothesized that the human AEO milieu was less proinflammatory than the murine AEO cultures; this interpretation was supported by snRNA-seq data showing only low level upregulation of RNA for key inflammatory cytokines, including TNF-α, IL-1β, IL-6, IP-10/CXCL10, and RANTES/CCL5 in human AEOs (Supplemental Figure 5). MRC5-derived feeder cells and the absence of an immune population resulted in expression of a smaller number of these proinflammatory cytokines, and at a lower level, than the murine primary feeder cells, suggesting that the absence of STEs in human AEOs may relate to an overall decrease in inflammatory signaling in non-epithelial cells following IAV infection. We can not, however, exclude the possibility that donor- or species-specific mechanisms could also be contributing to these differences in epithelial cell state given the high variance in human organoid cultures and fibroblast effects in published studies.

Human AEOs contained a large population of AT2 cells within each of the infection classes defined previously, so we used similar methods to classify Unexposed, Uninfected, Early, and Late Infected human AT2 cells (Figure 7, G and H). Consistent with IHC data, we noted more cells classified as Late Infected even at 8 hpi, with similar distribution at 16 hpi and a notable decrease in both infected Late Infected populations at 24 hpi, potentially suggesting a more rapid saturation of infection in human AEOs. We then performed DEG (Figure 7I) and differential chromatin accessibility analyses (Figure 7J) using the same analytical pipelines as used for the murine data. We noted similar dynamics of gene expression (Supplemental Figure 6) and chromatin accessibility in humans as in mice, with the largest changes notable comparing Unexposed to Uninfected AT2 driven by viral and immune response pathways. IAV infection led to a burst of translation-related pathways, consistent with viral replication driven by infection. This was followed by a substantial decrease in inflammatory pathways during Late infection, consistent with virus-mediated suppression as infection progresses (Supplemental Figure 4). These similar patterns implied a core conserved response to IAV across mammalian AT2 cells.

### Defining a core, evolutionarily conserved viral response in IAV-infected AT2 cells.

**Figure 8:**
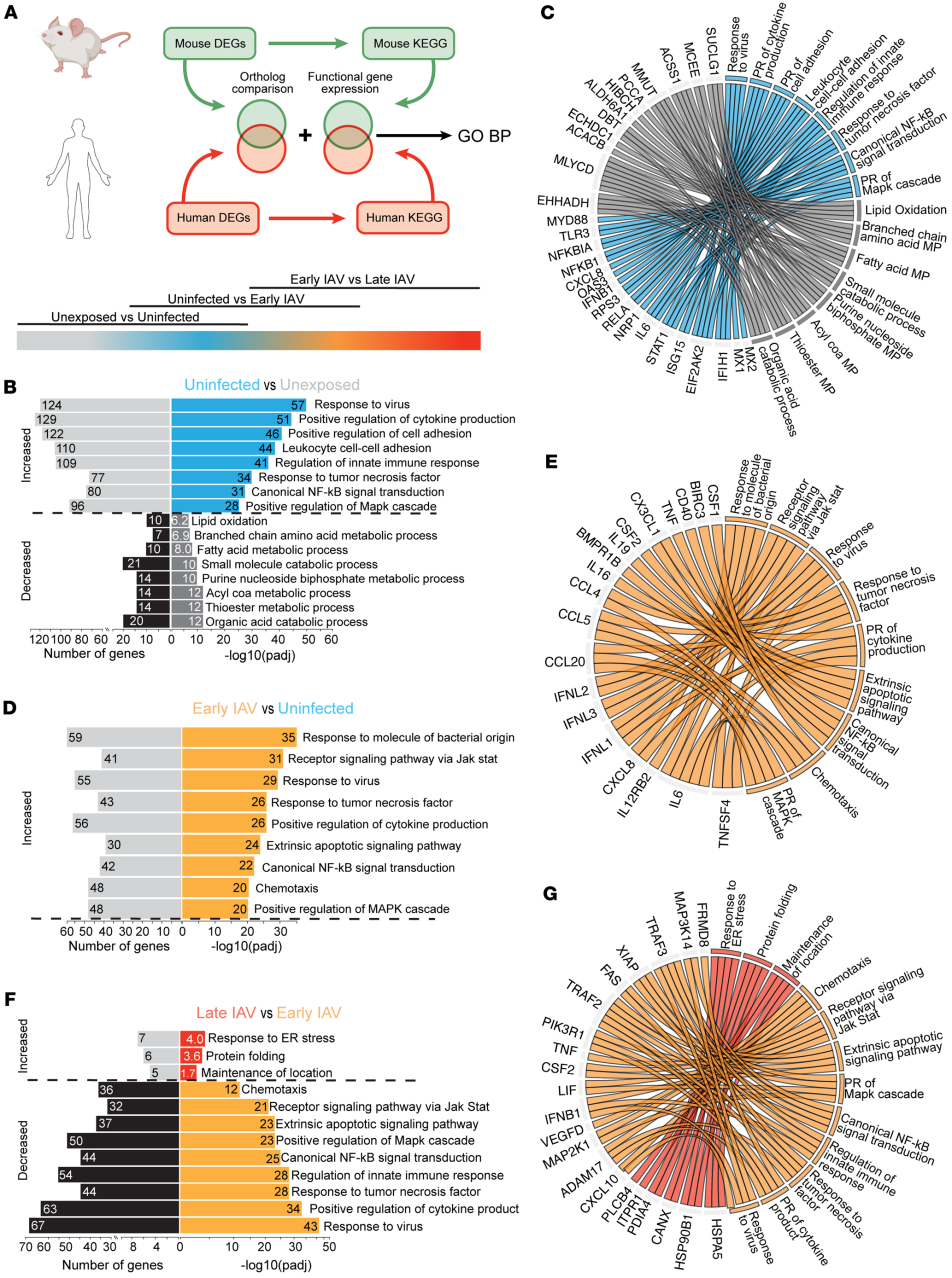
A conserved signature of IAV infection in AEOs. (**A**) Schematic depicting data analysis pipeline (see Results and Methods) used to identify core conserved regulated pathways and gene sets in IAV-infected AEOs comparing murine and human organoids from a 4-year-old donor. (**B** and **C**) Enriched and downregulated GO terms (**B**) in Unexposed versus Uninfected AT2, with leading-edge genes per GO terms in **C**. (**D** and **E**) Enriched GO terms (**D**) in Uninfected versus Early IAV AT2, with leading-edge genes per GO term in **E**. (**F** and **G**) Enriched and downregulated GO terms (**F**) in Early versus Late IAV infection in AT2, with leading-edge genes per GO term in **G**.

To identify this core antiviral response in AT2 cells after IAV infection, we developed a 2-step cross-comparison of the snRNA-seq results from the mouse and human AEOs to identify strongly conserved genes and pathways (Figure 8A). Using DEG signatures derived from pseudobulk expression profiles for each cell state (Unexposed, Uninfected, Early IAV, Late IAV) in both human and mouse, we performed pathway analysis and gene set enrichment analysis (GSEA) to identify conserved orthologs in the gene expression data (see Methods), and displayed GO pathway enrichment (Figure 8A and Supplemental Figure 7) that defined the AT2 response to IAV. We used stringent thresholds to avoid overinterpretation of sparse data and define a relatively small core conserved gene list for each stage of infection.

As expected, we found an early upregulation of genes associated with antiviral and proinflammatory signaling in Uninfected AT2, corresponding to the primed chromatin accessibility seen in this state in both species. GO BP output defined key pathways in viral response and immune activation (Figure 8B), with leading-edge genes that defined these signatures, including NFKB, TLR3, MYD88, STAT1, and ISG15 (Figure 8C and Supplemental File 1). Uninfected AT2 cells downregulated metabolic pathways necessary for surfactant metabolism, including fatty acid β-oxidation and lipid catabolic processes (Figure 8B), with these signatures defined by a decrease in critical metabolic enzymes, including ACACB and PCCA. Immunofluorescent staining of murine AEOs confirms loss of SPC staining in NP^+^ infected cells early, with increasing NP^–^ AT2 SPC loss later (Supplemental Figure 8A).

Comparing Early infection to Uninfected AT2, we noted no downregulated pathways and found that 87% of pathways enriched in Early infection were present in the Uninfected signature but further upregulated following infection initiation (Figure 8, D and E). The remaining 13% of pathways and leading-edge genes included those associated with viral infection, with an increase in vesicle trafficking, viral assembly, and budding, as well as regulation of cell death. At the Late IAV stage, we noted upregulation of ER stress and protein folding response pathways, with leading-edge genes including chaperones HSPA5 and HSP90B1 and the critical ER regulator CANX. Antiviral pathways were broadly downregulated, suggesting conservation of virus-mediated immune suppression in late infection across both murine and human AT2 cells. These trends are defined by decreased expression of previously upregulated factors from the primed state, including NF-κB, MAPK, IFN, apoptotic signaling, and other immune cell mediators (Figure 8, F and G).

## Discussion

Here, we present data from temporal modeling of IAV infection in murine and human AEOs. These data provide both a resource of transcriptomic and epigenomic mechanisms of IAV pathogenesis in AT2 cells and a technical advancement with defined protocols for viral infection and analysis in primary lung organoids. Organoid technology has enabled substantial advancement in regeneration and genetic disease. To date, however, most infection in lung organoids (24, 25, 29, 32, 33) has been primarily proof of principle, designed to verify cellular state in a new organoid method rather than disease modeling. Our data move beyond proof of principle to define a core signature of infected AT2 cells, including loss of surfactant processing (58) and an active response to inflammatory milieu prior to infection to prime for antiviral response in early infection (31). We also define a distinct shift in cellular metabolism from lipid and amino acid metabolism towards glycolysis and carbohydrate metabolism (59, 60), a metabolic change critical for viral pathogenesis (61). Each of these characteristics mirror an important aspect of IAV-driven ARDS pathology and demonstrate that the impact of IAV in the alveolus is not solely a product of infected cells, but also an epigenomic shift in bystander AT2 cells presumably in an attempt to survive in the infected milieu. When these early responses fail, IAV infection leads to hijacking of host machinery, which is recruited to translate viral proteins, resulting in increased ER stress and an upregulation of ribosomal pathways while immune signaling is suppressed. These data define key targets associated with each aspect of IAV viral pathogenesis in AT2 cells and demonstrate that critical aspects of IAV pathogenesis and host response are conserved in AEOs.

Our data also demonstrate the capacity for uninfected AT2 cells to lose their AT2 cell identity and transition into a damage-associated STE state similar to previously established models of stressed and transitional lineages in the lung. Notably, these cells were detected in our murine model but not our human AEOs and appear to be a product of the inflammatory milieu rather than direct infection. This interpretation is supported by the absence of markers of IAV infection, including Isg15 and Oas1 (62), and the absence of viral transcripts in STEs in murine AEOs. Human AT2 cells are known to enter into STE-like states following diverse injuries, including IAV (44), so it is of interest that they do not do so in our human AEOs. We observed a less proinflammatory milieu in human AEOs, which we speculate may be due to (a) differential fibroblast activation in primary murine mesenchyme compared to MRC5 fetal human cells, (b) absence of an immune population and reduced cytokine production in human AEOs compared with murine, or (c) a young human lung donor who may have increased resilience to viral injury at the cellular level. Furthermore, the role of STEs in pediatric lungs following IAV infection or other injuries is unclear, which may also have contributed to this finding. A limitation of our study is the presence of a single human donor for human AEOs, but multiple biological replicates and pooled temporal sequencing results resulted in a large pool of AT2 cells for study with a controlled genetic background. A key goal of the combinatorial analysis of human and mouse was to use the human signature to introduce potentially conserved aspects of the more robust murine organoid modelling, allowing us to maximize the impact of the data resource without overinterpreting data from a single human donor. Our methodological advances may provide a lower barrier of entry for future studies focused on understanding the possible diversity of IAV responses in AT2 cells present across the human population.

A provocative aspect of our study is the extensive epigenomic consequences for AT2 cells within an IAV-induced inflammatory milieu. Previous data had implied a key role for flu-specific gene expression in driving epigenomic changes in infected cells, but we were surprised to find the most substantial changes to chromatin accessibility occurring due to inflammatory signaling in uninfected cells. Our data suggest this change is driven by immune response signals from the milieu and is conserved in both mouse and human AEOs exposed to IAV. We speculate that this rapid epigenomic priming functions to prepare cells for a robust antiviral response following infection and is stimulated by the immune milieu when virus is detected by the host. This progression is conserved in mouse and human AT2 cells, implying potential therapeutic benefits for the host. Our data are also consistent with a model of epigenomic dysregulation in infected cells, notably the apparently random chromatin opening observed in early IAV infection, which may result from readthrough transcription (17). While some IAV-infected pulmonary cells survive infection and persist in regenerating tissue (63, 64), AT2 cells that survive influenza B infection have reduced regenerative capacity (64) and many infected cells are cleared by the immune system or die following virus-mediated cell lysis. Our data suggest that targeting the response of bystander cells may provide an orthogonal approach to improving alveolar resilience during viral pneumonia.

Furthermore, it is tempting to speculate that this major impact on bystander AT2 function may underlie part of the limited impact of existing IAV treatments, including neuraminidase inhibitors (NAIs) such as Tamiflu (oseltamavir). Although effective at reducing symptoms when given within the first 48 hours following clinical presentation, NAIs are less effective when given later (17) or administered in cases of severe infection (65). This decreased effectiveness later in disease is poorly understood, and our data imply that some aspects of viral pneumonia and ARDS may occur due to the immune response in uninfected AT2 cells. It is possible that suppression of infected cells may be insufficient for symptomatic benefit after a certain period of immune activation. Given that IAV-induced ARDS remains a global clinical challenge with few effective interventions, the cellular resolution provided in our studies may provide a path forward to new therapies. AEO model systems, including our own, are highly tunable for applications such as screening for IAV therapeutics or genetic modifiers of AT2 response (28), enabling ongoing translation-focused studies in lung biology. Although there is still much to be learned about IAV-host interactions, future studies in primary organoid models can provide flexible, reductionist systems to identify mechanisms underlying the cellular and physiological disruptions induced by viral pathogens that drive progression to ARDS.

## Methods

Many of the methods used for organoid preparation and staining in this manuscript have been previously described (24).

### Sex as a biological variable.

All mouse AEO experiments included cultures derived from both male and female mice. Human results include AEOs obtained from lung tissue from a single 6-year-old female donor.

### Mice.

Mouse lines used included C57BL/6J mice (Jackson Laboratory, strain 000664), Axin2^creERT2-TdT^ (a gift from Edward Morrisey, University of Pennsylvania, Philadelphia, Pennsylvania, USA) (15, 66), and R26R^EYFP^ (B6.129 × 1-Gt(ROSA)26Sor^tm1(EYFP)Cos^/J; Jackson Laboratory, strain 006148) (67).

### Mouse PR8 (Cre) infection.

Mixed background mice containing R26R^EYFP^ were weighed and administered 0.4 hemagglutinating units per gram of PR8 H1N1 influenza containing Cre recombinase (63, 64) (a gift from Nicholas Heaton and Emily Hemann, Ohio State University, Columbus, Ohio, USA) at 8–12 weeks of age. After 48, 72, and 96 hours, mice were weighed again and lung tissue was harvested as below.

### Mouse lung harvest for single-cell suspension.

Mice were anesthetized via isoflurane exposure, followed by euthanasia via cervical dislocation and thoracotomy. The chest cavity was opened to expose the heart and lungs. The right ventricle was perfused with 5–10 mL of cold PBS to clear blood from the lungs. The perfused lungs were removed, and the individual lobes were isolated, removing as much airway as possible, and placed in cold PBS on ice. Single-cell suspensions were generated as previously described (24).

### Flow cytometry of mouse lungs.

Single-cell suspensions were obtained as above, and cells were resuspended in 5 mL MACS buffer (autoMACS Rinsing Solution [Miltenyi Biotec, 130-091-222] with MACS BSA Stock Solution [Miltenyi Biotec, 130-091-376]) and passed through a 40 μm filter (Greiner Bio-One, 542040). Cells were centrifuged and the cell pellet was resuspended in Fc Receptor Binding Inhibitor Polyclonal Antibody (Invitrogen, 14-9161-73) diluted 1:100 in MACS buffer and incubated for 10 minutes at room temperature. Following centrifugation, cells were resuspended in 100 μL antibody cocktail (see Supplemental Tables 1 and 2 for details) consisting of Podoplanin-BV421, CD31–eFluor 450, MHC-II–BV480, CD326/EPCAM-BV785, CD24–PE-Cy7, ItgB4/CD104–Alexa Fluor 647, CD45–Alexa Fluor 700, Fixable Viability Dye eFluor 780, and SNA-FITC. Single-color controls were generated in 1.5 mL Eppendorf tubes using control cells aliquoted from vehicle/uninfected mice or, in the case of the Pr8 Cre experiment, from flu-infected mice prior to staining. Cells and controls were washed and resuspended in MACS buffer and passed through a 35 μm filter lid (Corning, 352235). A Cytek Aurora was used for the data collection and spectral unmixing with FloJo for subsequent analysis.

### Murine epithelial processing for AEOs.

Single-cell suspensions were obtained as above, and cells were resuspended in 5 mL MACS buffer in a mixture of the following antibodies diluted 1:100 in MACS buffer and incubated for 10 minutes protected from light: CD31–eFluor 450, CD45–eFluor 450, CD326-APC, and Fixable Viability Dye eFluor 780. Cells were washed and prepared for sorting as above. Using single-stain controls from experimental animals and wild-type littermates (TdTomato^−^) for compensation and adjusting gating to remove debris/doublets, the live/CD31^−^CD45^−^CD326^+^(EpCAM^+^)TdTomato^+^ (Wnt-active AT2) population was sorted into complete SAGM.

### Fibroblast stock preparation and maintenance.

Primary lung fibroblast stocks were generated from P28 C57BL/6 mice as previously described (24). For the use of frozen fibroblast stocks in AEOs, 2–4 days prior to use in AEOs, cells were thawed and replated in 10 cm plates in fibroblast media. On the day of AEO culture, fibroblasts were washed, trypsinized, resuspended, and counted prior to addition to culture. For CD45^+^ cell–depleted fibroblast experiments, details of CD45^+^ cell depletion via bead sorting prior to plating were reported previously (24). Maintenance, passaging, and freezing continued as for the non-depleted fibroblasts.

### AEO growth.

To generate “spiked” SAGM for mouse AEOs, SABM (Small Airway Epithelial Cell Growth Basal Medium; Lonza, CC-3119) was combined with the following additives: SAGM SingleQuots Supplements and Growth Factors (using only the BPE [2 mL], insulin [0.5 mL], retinoic acid [0.5 mL], transferrin [0.5 mL], and hEGF [0.5 mL] aliquots) (Lonza, CC-4124), Heat-Inactivated Fetal Bovine Serum (Corning, 35-011-CV, final concentration 5%), Antibiotic-Antimycotic (Gibco, 15240-062, final concentration 1×), and Cholera Toxin from *Vibrio cholerae* (Sigma-Aldrich, C8052, final concentration 25 ng/mL). For human cultures, T3 and hydrocortisone (0.5 mL each) were added to spiked SAGM. AEOs were generated as previously described (24) and grown in a mixture of 50% Matrigel/50% SAGM on a 0.4 μm Corning Transwell insert in a 24-well plate. Media were changed every 48 hours.

### Infection medium.

To generate infection medium for mouse AEOs, SABM (Lonza, CC-3119) was combined with the following additives: SAGM SingleQuots Supplements and Growth Factors described as above for the spiked SAGM. TPCK-treated trypsin from bovine pancreas (Sigma-Aldrich, T1426) was added to a final concentration of 2 μg/mL.

### AEO infection.

AEOs for flu and controls were resuspended in 300 μL/well spiked SAGM. BSA precoating of pipette tips was not needed due to the presence of serum in the media. PR8 IAV (1.025 × 10^6^ PFU; Charles River) was added per 300 μL SAGM or per well of AEOs. Organoids were then moved to an Ultra Low Adhesion Plate for incubation at 37°C. At each time point, 1 mL of 4% paraformaldehyde was added to each well and the plate incubated at 4°C for 45 minutes. AEOs of like condition were pooled in 15 mL conicals and spun down at 100*g* for 5 minutes at 4°C before proceeding to permeabilization.

### Human AT2 cell isolation.

Distal lung tissue grossly assessed as healthy for culture was isolated to avoid airway and dissected into 0.3 g portions in 10 cm dishes containing PBS with 1× Antibiotic-Antimycotic. As with the mouse lung, these portions were placed into a GentleMACS C tube and cut into small pieces before adding 5 mL Digestion Buffer. Samples were digested and washed as above and passed through a pre-rinsed 100 μm filter (Greiner Bio-One, 542000). The samples were centrifuged at 800*g* for 5 minutes at 4°C and resuspended in 5 mL RBC lysis buffer. This suspension was then filtered through a pre-rinsed 40 μm filter and spun at 500*g* for 5 minutes at 4°C. The supernatant was removed, and the cells were resuspended in 500 μL of 1:10,000 FITC LysoTracker (Cell Signaling Technology, 8783S) in MACS buffer for 30 minutes at room temperature. In the last 10 minutes, 5 μL Fc receptor block in MACS buffer was added to the suspension. Ten milliliters of MACS buffer was added and the cells spun at 500*g* for 5 minutes at 4°C. Cells were resuspended in an antibody cocktail including EpCAM (Invitrogen, 12-9326-42) and Fixable Viability Dye eFluor 780 for 10 minutes on ice. Single-stain controls were generated from unstained cells aliquoted before and after LysoTracker staining and before the antibody cocktail. After rinsing once with 10 mL, the cell pellet was resuspended in MACS buffer (volume adjusted for cell count) and passed through a 35 μm filter lid into a FACS tube for sorting. The Live CD326^+^(EpCAM^+^)LysoTracker^+^ (AT2) population was sorted into a tube containing spiked SAGM for human cells (see above) at 4°C, using a BD FACSAria Fusion cell sorter with a 100 μm nozzle.

### Human AEO culture.

Human AT2 cells were plated as described above for murine AT2 except MRC5s (ATCC, CCL-171; tested negative for mycobacterial contamination) at P8 were used as a replacement for the murine fibroblast stock. Human AT2s were then plated at 1:10 ratio with MRC5 in 50% Matrigel/50% SAGM.

### Isolation of AEOs for infection or whole-mount immunofluorescence.

Transwells were washed (above and below) with 500 μL of ice-cold PBS. Then, 500 μL of Cell Recovery Solution (Corning, 354253) was added to each Transwell, and a wide-bore pipette tip was used to mechanically disrupt the Matrigel; the mixture was pipetted up and down and transferred to a new Ultra-Low Adhesion 24-well plate (Corning, 3473). AEOs were washed and stained as previously described (24). When staining with SNA-FITC, blocking was done per manufacturer’s instructions using Carbo-Free Blocking Solution, 10× Concentrate (Vector Laboratories, SP-5040). Isolated AEOs then underwent IHC, as previously described using the antibodies in Supplemental Table 1, followed by clearing in fructose-glycerol clearing solution (60% vol/vol glycerol + 2.5 M fructose) and mounting. Slides were imaged immediately or stored at 4°C.

### ELISA analysis.

Immediately prior to liberation of AEOs for nuclei isolation, 50–100 μL of suspension media was collected from AEO samples. These samples were analyzed with a custom Luminex panel in conjunction with the Research Flow Cytometry Facility at Cincinnati Children’s Hospital Medical Center (CCHMC) following the manufacturer’s protocol.

### AEO dissociation and preparation of single-cell suspension for scRNA-seq and scATAC-seq.

Transwells were washed (above and below) with 1 mL of PBS. Then, 60 μL of organoid digest buffer (Dispase [Corning, 354235, undiluted, 50 U/mL], DNase I [GoldBio, D-301, final concentration 5 U/mL], Collagenase Type I [Gibco, 17100017, final concentration 4,800 U/mL)] was added, and Matrigel plugs were gently disrupted. Nuclei were obtained following washing and digestion as previously described (24).

### Sequencing/library preparation.

From each condition, a single-nucleus preparation was prepared as described above, and a maximum of 16,000 nuclei were loaded into a channel of a 10X Genomics Chromium system by the CCHMC Single Cell Genomics Facility. Libraries for single-nucleus multiome sequencing were generated following the manufacturer’s protocol. Sequencing was performed by the CCHMC DNA Sequencing Core using Illumina reagents. Raw sequencing data were aligned to the mouse reference genome mm10 (35) or human reference hg38 (68) with CellRanger-arc 2.0.2 (69) to generate expression count matrix files. To detect flu genes, a contig for each positive and negative PR8 IAV gene strand was added to each genome following 10X Genomics “Build a Custom Reference” instructions.

### Data QC and analysis.

After alignment, ambient RNA contamination was addressed using single-cell contamination detection and correction (scCDC) (38), which identified contamination genes using a 0.4 restriction factor, quantified contamination ratios, and generated corrected count matrices. Using Seurat (70), RNA parameters from the multiome object were used to filter cells and to retain those with 300–3,500 detected features and <50% mitochondrial gene expression. Mitochondrial genes and MALAT1 were removed from downstream analysis. Doublet detection was performed using DoubletFinder (37) with optimized pK parameters determined through parameter sweeping, and only singlet cells were retained for analysis. For snATAC-seq data, Signac (69) was used to filter cells based on ATAC fragment counts (1,800–100,000), TSS enrichment (>2), nucleosome signal (<1), and blacklist region overlap (<5%). Data integration was performed using Harmony (71) to correct for batch effects across samples, followed by weighted nearest neighbor (WNN) analysis combining both RNA and ATAC modalities. The final dataset included cells from 6 samples across 3 time points (8, 16, and 24 hours) under control and flu treatment conditions. Clustree (72) and clusterProfiler (73) were used for cluster identification.

### Statistics.

Throughout the study, 2 groups were compared by 2-tailed *t* test and groups of 3 or more groups were compared by 3-way ANOVA with prespecified multiple comparison testing. For sequencing data, RNA analysis was described above. Briefly, we pseudobulked the AT2 cells by infection stage with equal distributions of each time point and then used GSEA with DESeq2 (74) Wald statistics to determine DEGs. These were then filtered to include only a log_2_FC of greater than 0.38 and an adjusted *P* value of less than 0.05. To analyze the snATAC-seq data, we used an ArchR-inspired (49) approach that took differential peak results identified by DESeq2 as input, linked peaks to genes, and then utilized ArchR’s exponential decay function and distance weighting approaches for gene activity scoring with gene size normalization. The top 25% of active (highly active) genes from these data were used for ORA analysis, with the remaining significantly changed genes serving as the detection universe for analysis. All GO BP analyses were done by filtering to pathways between 5 and 300 genes in size, with results then filtered based on an adjusted *P* value of less than 0.05 following FDR correction using the Benjamini-Hochberg method. For human to mouse comparison, we performed Kyoto Encyclopedia of Genes and Genomes (KEGG) pathway enrichment analysis using GSEA with DESeq2 Wald statistics as the ranking metric for each species, and generated enriched pathways for each of our infection state comparisons. KEGG pathways for each comparison were identified per species, and overlapping pathways between species were identified based on KEGG IDs. Genes contributing to the overlapping pathways for each comparison with the same directional enrichment were then used to create a gene list containing mouse and human genes that contributed to the same pathways and thus similar functional outcomes. Shared orthologs between each species’ DEGs were added to this core gene list, and the final conserved gene list was analyzed using GO BP to determine a final conserved activity profile between each stage of infection in AT2 cells. For box and whisker plots, the box shows the 25th to 75th percentile of the distribution, the line shows the mean, and the whiskers show the minimal and maximal values.

### Study approval.

All animal studies were conducted under the guidance and supervision of the CCHMC Institutional Animal Care and Use Committee (IACUC) in accordance with CCHMC regulatory protocols. For human studies, de-identified postmortem primary human lung tissue was obtained from the local Cincinnati organ procurement organization in accordance with University of Cincinnati College of Medicine (UCCOM)/CCHMC regulatory guidelines. While this research is not considered human studies research based on the 2018 NIH Common Revised Rule definition of Human Studies Research, tissue was accepted under UCCOM IRB no. 2013-8157 (for which WJZ is a registered investigator) following approved protocols. Tissue for this study was donated by the family of a single 6-year-old female donor with a head injury that was not used for clinical transplantation due to a transient drop in oxygen saturation immediately prior to collection.

### Data and materials availability.

All mouse and human organoid snRNA-seq and snATAC-seq data have been deposited in the NCBI Gene Expression Omnibus (GEO GSE327614) and on the NIH LungMAP portal at http://www.lungmap.net Enrichment data used to generate all figures are available as a Supporting Data Values file.

## Author contributions

Conceptualization – AE and WJZ. Data acquisition – AE, SF, HIWN, BZ, AT, and KCSK. Data analysis – AE, BZ, ALZ, and WJZ. Supervision – WJZ. Writing – original draft – AE and WJZ. Writing – review and editing - all authors.

## Conflict of interest

The authors have declared that no conflict of interest exists.

## Funding support

This work is the result of NIH funding, in whole or in part, and is subject to the NIH Public Access Policy. Through acceptance of this federal funding, the NIH has been given a right to make the work publicly available in PubMed Central.

- National Institute of General Medical Sciences grant GM063483 (to AE, BZ, and AT).
- National Heart, Lung, and Blood Institute grants HL007752 (to BZ, HIWN, and AT) and HL166245, HL140178, and HL156860 (to WJZ).
- National Institute of Allergy and Infectious Diseases grant AI150748 (to WJZ).

## Supplementary Material

Supplemental data

Supplemental data set 1

Supporting data values

## Version 1 07/02/2026

In-Press Preview

## Version 2 08/10/2026

Electronic publication
