# Supplementary material for: Early cell-autonomous and niche-mediated epithelial response to influenza infection in primary alveolar organoids
Source: JCI Insight. 2026 Jul 2;11(15):e201739. doi: 10.1172/jci.insight.201739 (PMC13464008; doi:10.1172/jci.insight.201739)
Supplement: Supplementary file 1 [file jciinsight-11-201739-s271.pdf]

SUPPLEMENTAL MATERIALS

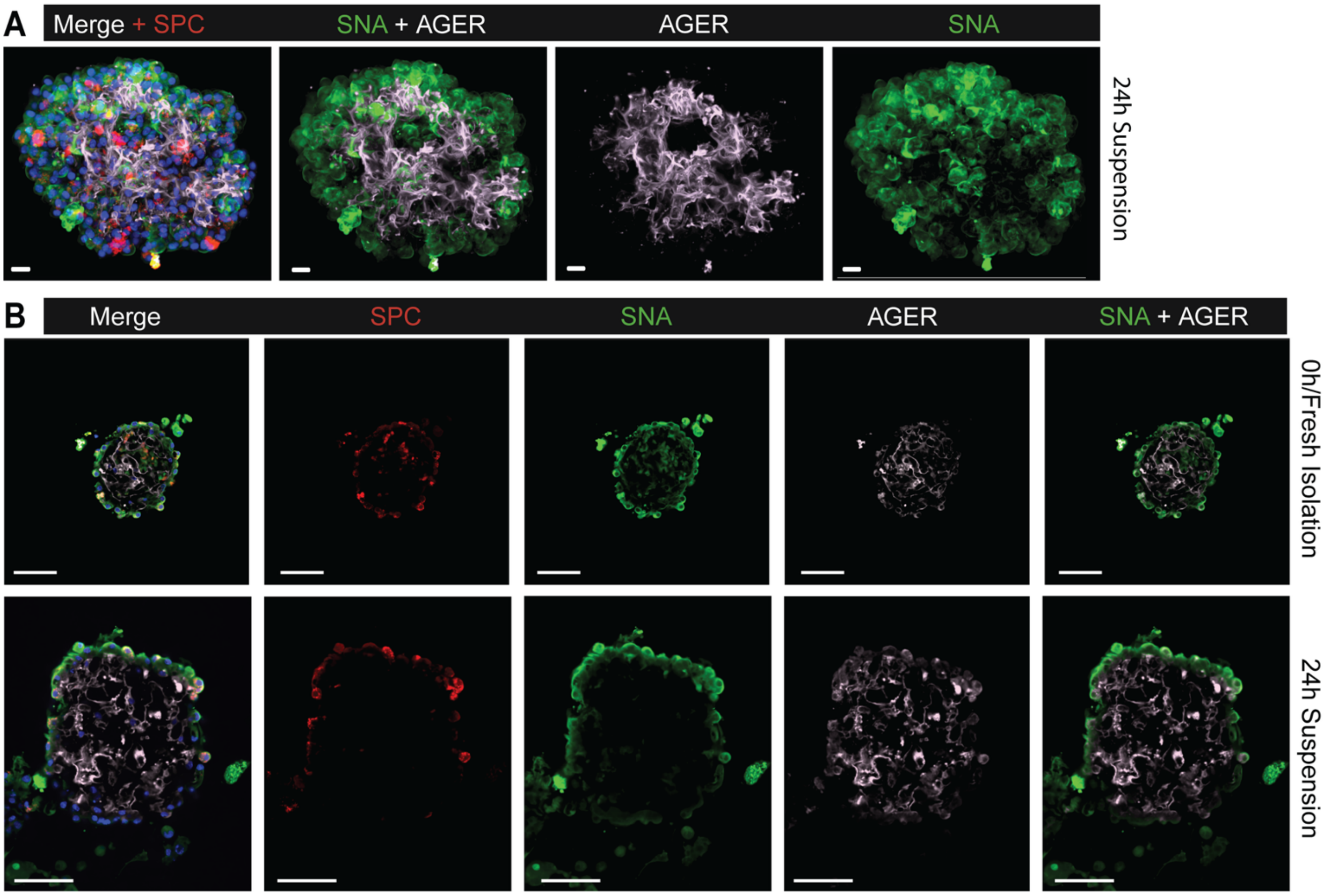

Supplementary Figure 1. α2,6 sialic acid polarity is conserved in suspension. (A) Expression of the flu binding a-2,6-sialic acid in AEOs marked by expression of sambuccus nigra lectin predominantly in AT2 cells. (B) α2,6 sialic acid expression (SNA) in organoids immediately upon removal from well compared to an organoid suspended in SAGM for 24 hours shows no notable change in cell polarity. Scale bars = 50μm

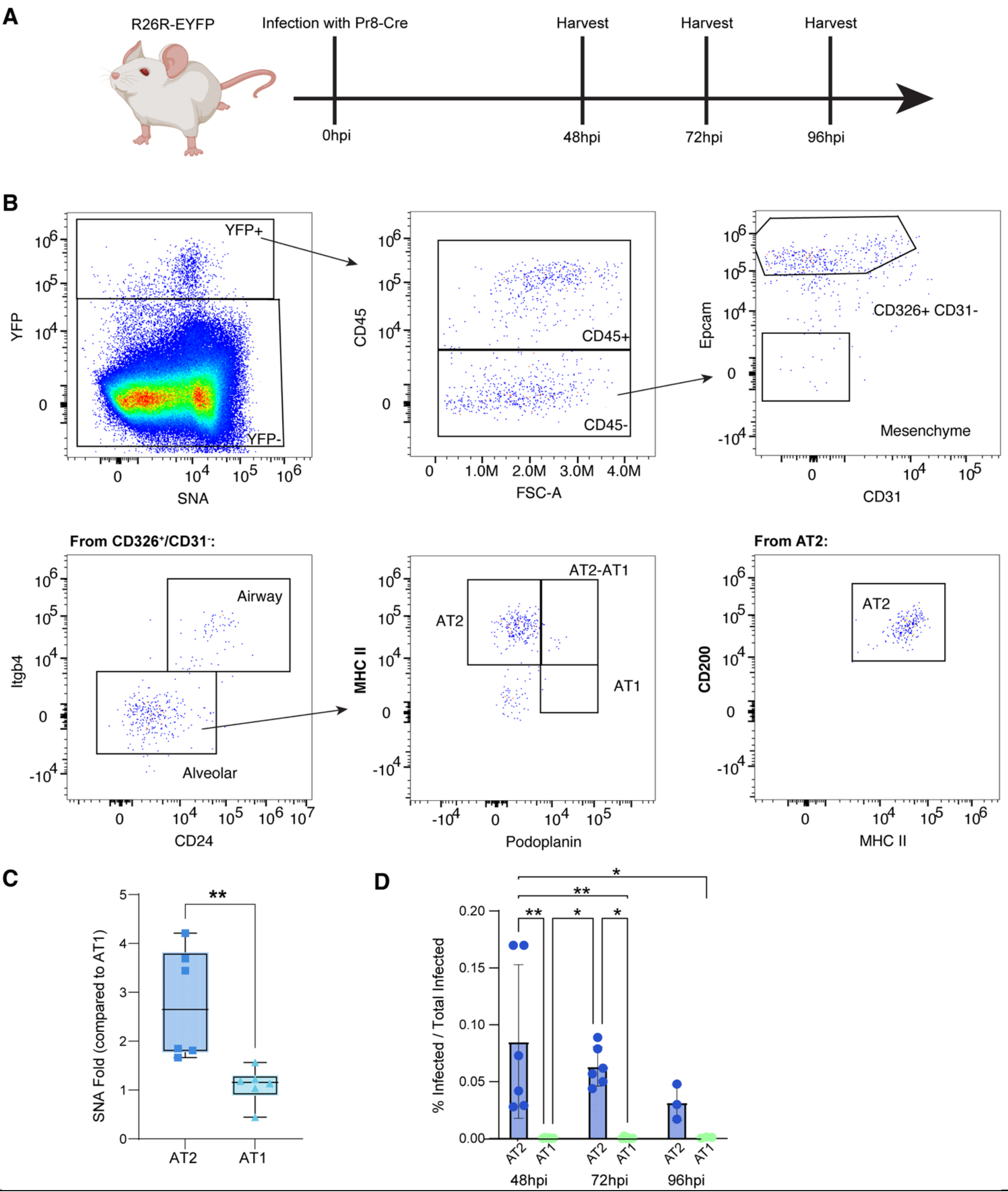

Supplementary Figure 2. PR8-Cre infection initiation experiment and flow gating. (A) R26R^{EYFP} mice were infected with a Pr8-Cre virus and then harvested for flow cytometry 48, 72, and 96hpi. (B) Flow cytometry gating showing the identification of our YFP^{+}/IAV-Infected cells with a predominance of infected AT2 cells relative to other populations. (C-D) *In vivo* confirmation of SNA expression (C) and increased infection rate (D) in AT2 cells during early flu via flow cytometry.

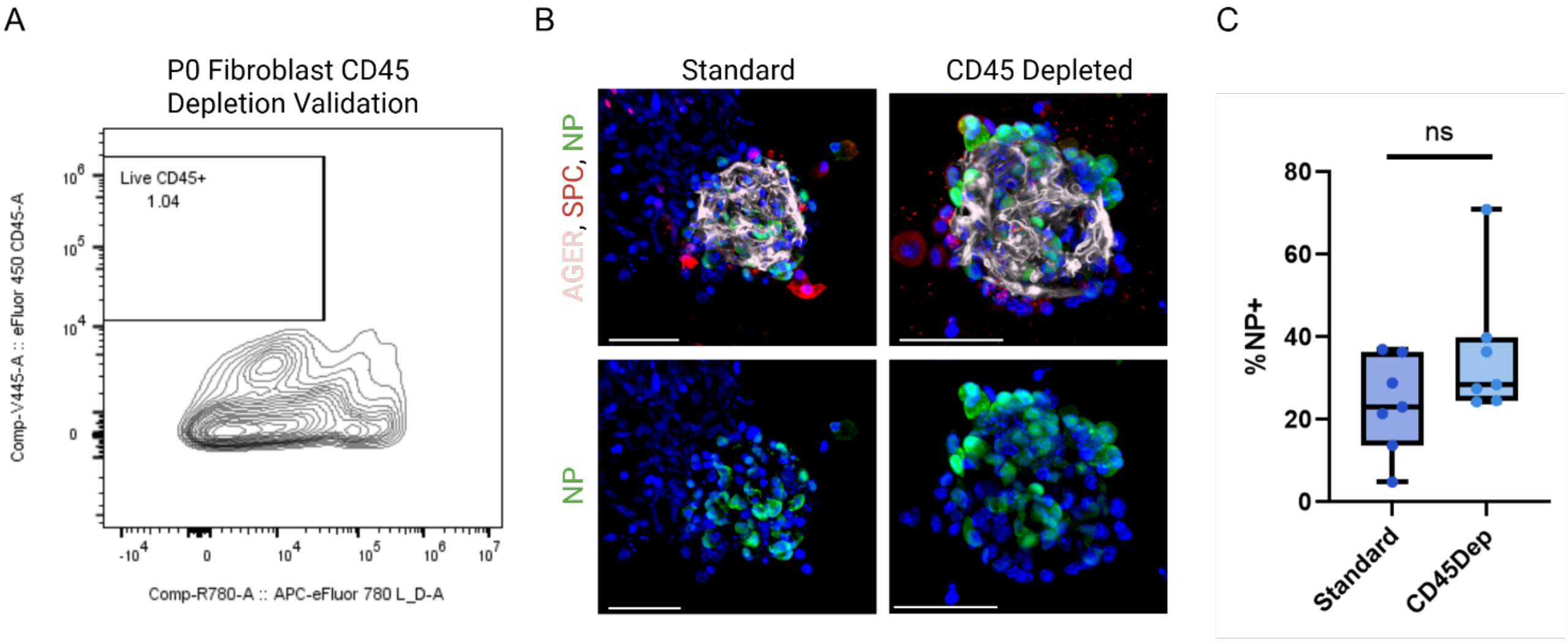

Supplementary Figure 3. Immune population depletion in feeder culture does not affect infection kinetics. (A) Flow cytometry gating showing successful depletion of $CD45^+$ immune population. (B) IF of 24hpi organoids and (C) quantification of NP^+ IAV-infected cells shows no change in IAV infection kinetics without an immune population. Scale bars = 50µm

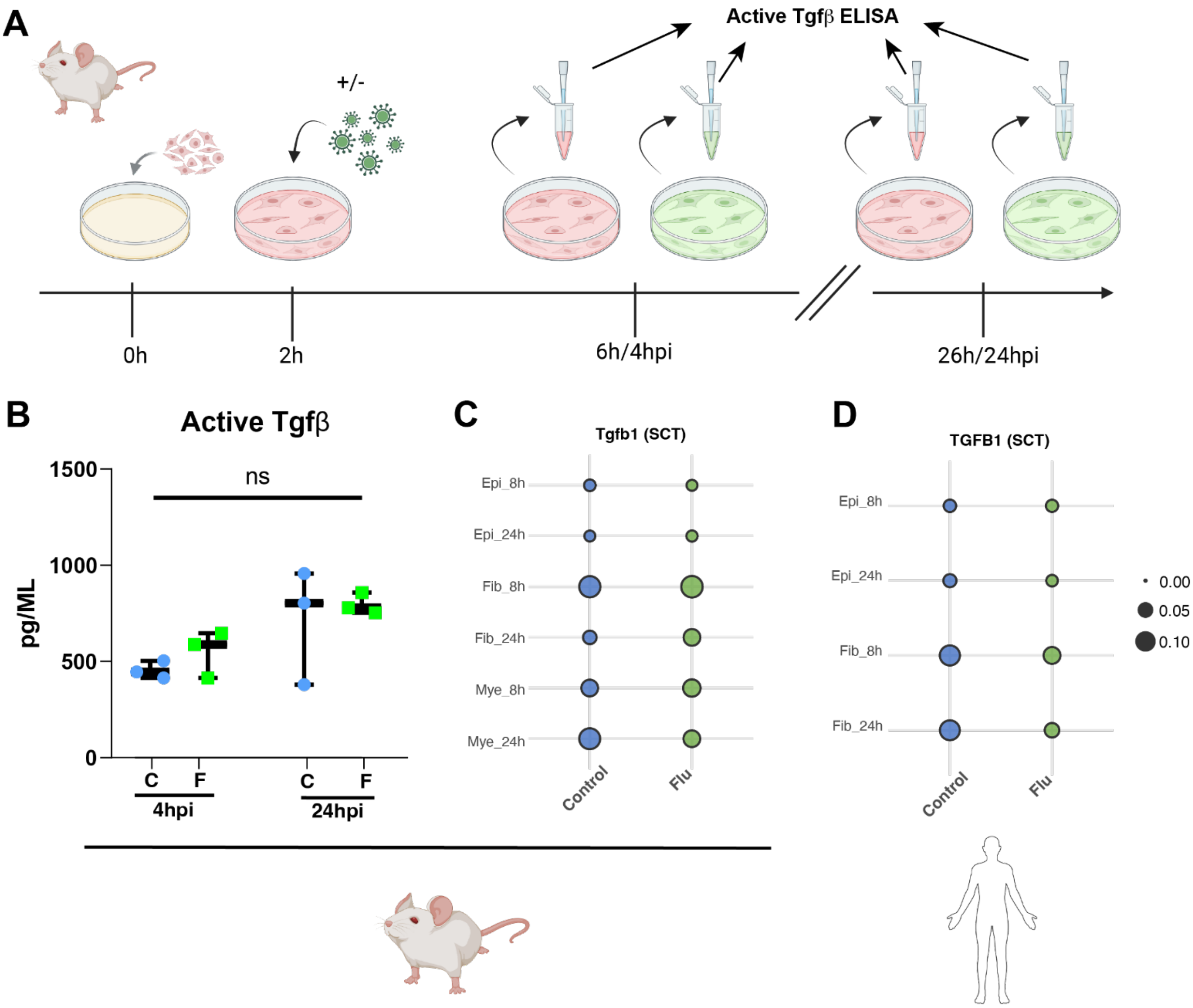

Supplementary Figure 4. TGFβ activity and expression in the IAV-exposed milieu. (A) Schematic of D21, P3 mouse lung fibroblasts plated then cultured with or without 1.025 PFU/100k cells IAV for up to 24hpi with culture supernatant samples removed at 4 and 24hpi from IAV-exposed and control wells. (B) ELISA of culture supernatant shows an insignificant increase in active Tgfβ expression between the 4 and 24hpi timepoints and no change from control cultures. (C) Tgfb1 expression in murine AEO transcriptome data shows no difference between flu and control mean expression at 8 or 24hpi. (D) Human AEO TGFB1 expression shows no change in mean transcript expression in 8 or 24hpi.

Supplementary Figure 5. Human gene expression and Mouse ELISA and gene expression of key inflammatory cytokines. (A) ELISA data from 8 and 24hpi and unexposed AEO supernatant samples of key inflammatory cytokines, adjacent to normalized murine AEO RNA expression* within major populations (Epi = Epithelial, Fib = Fibroblast, Mye = Myeloid) reveals conserved trends between detectable protein levels and transcriptional regulation. (B) Transcription data from human AEOs indicates a unique expression signature distinct from the murine AEO culture, suggesting cellular cross-talk in the niche is critical for regulating immune signaling response. *All use assay as indicated except for Interferon alpha which was analyzed based on expression of available genes. For mice, Ifna2 and Ifna4 were detected while IFNA2 and IFNA5 were detected in the human samples for scoring. Scale for dots representing mean expression for both mouse and human data is provided to the right of each plot. P values by ANOVA with multiple comparisons: * < 0.05, ** < 0.01, *** < 0.001, **** < 0.0001. (see next page).

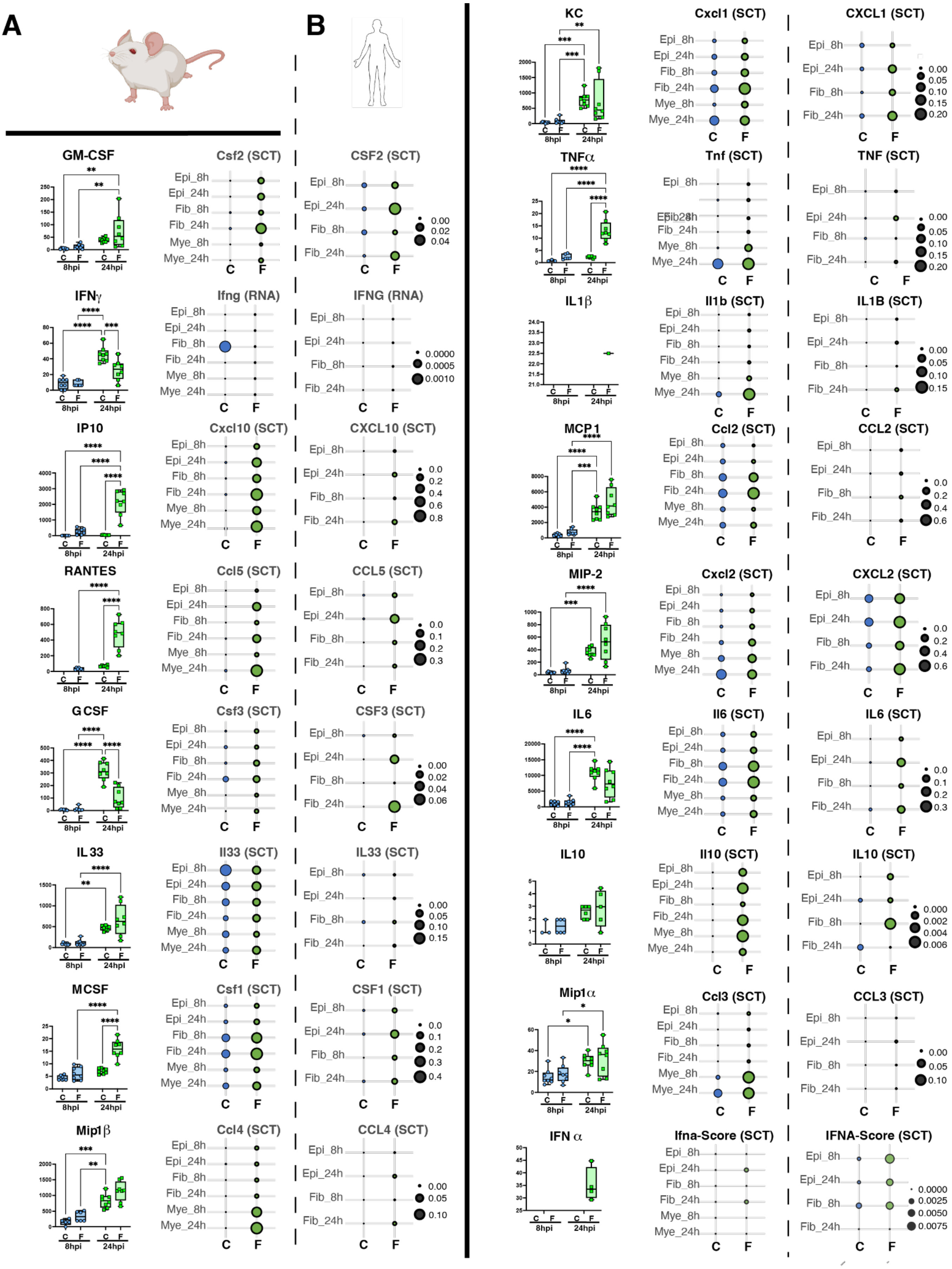
A
B
GM-CSF
Csf2 (SCT)
CSF2 (SCT)
KC
Cxcl1 (SCT)
CXCL1 (SCT)
IFNγ
Ifng (RNA)
IFNG (RNA)
TNFα
Tnf (SCT)
TNF (SCT)
IP10
Cxcl10 (SCT)
CXCL10 (SCT)
IL1β
Il1b (SCT)
IL1B (SCT)
RANTES
Ccl5 (SCT)
CCL5 (SCT)
MCP1
Ccl2 (SCT)
CCL2 (SCT)
G CSF
Csf3 (SCT)
CSF3 (SCT)
MIP-2
Cxcl2 (SCT)
CXCL2 (SCT)
IL 33
Il33 (SCT)
IL33 (SCT)
IL6
Il6 (SCT)
IL6 (SCT)
MCSF
Csf1 (SCT)
CSF1 (SCT)
IL10
Il10 (SCT)
IL10 (SCT)
Mip1β
Ccl4 (SCT)
CCL4 (SCT)
Mip1α
Ccl3 (SCT)
CCL3 (SCT)
IFN α
Ifna-Score (SCT)
IFNA-Score (SCT)
Epi_8h
Epi_24h
Fib_8h
Fib_24h
Mye_8h
Mye_24h
C
F
8hpi
24hpi

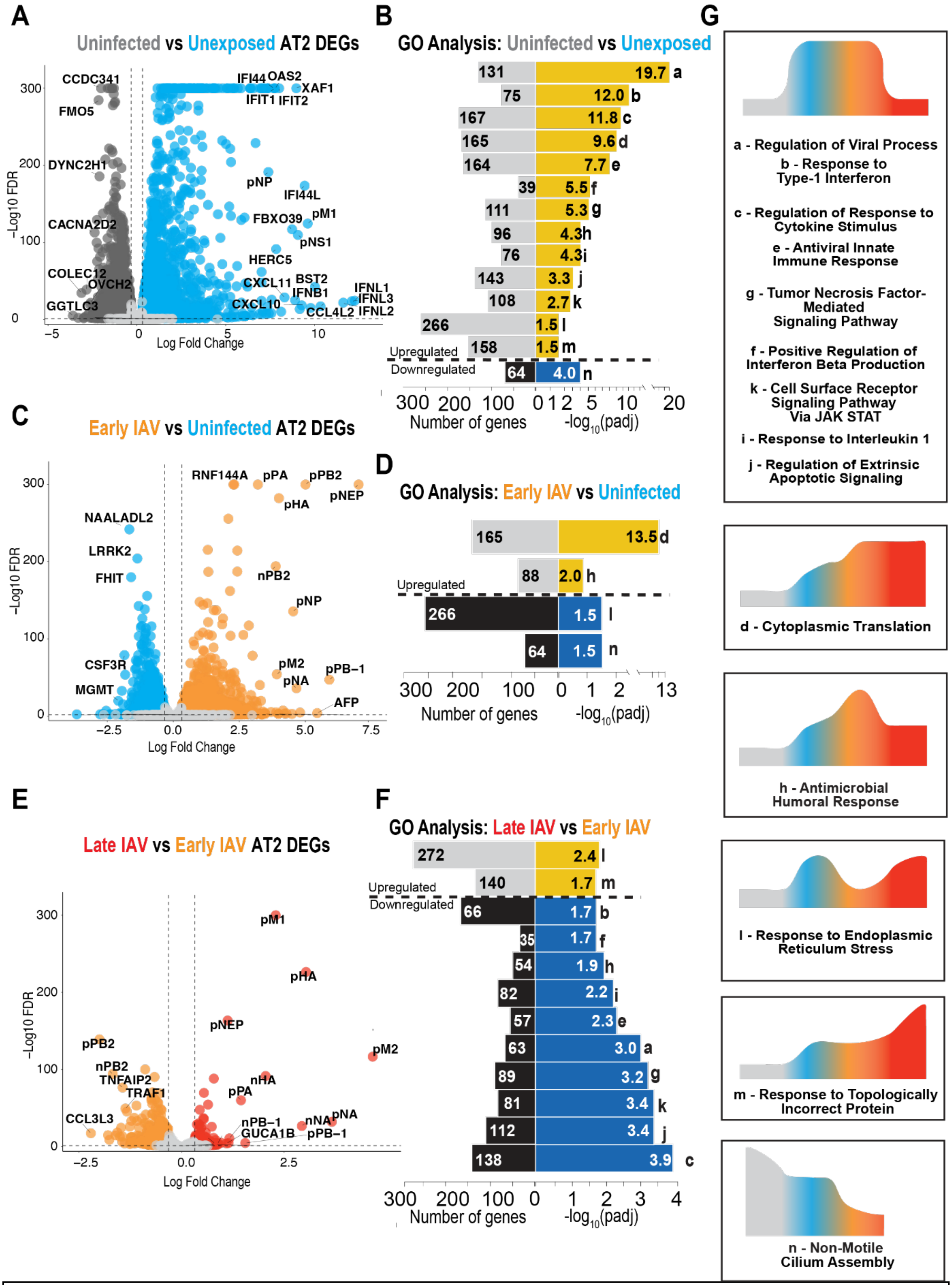

Supplementary Figure 6. Human AT2 transcriptome analysis with GO-BP trends. (A-B) Volcano plot of DEGs (A) and GO Biological Process enrichment (B) of genes in Unexposed (grey) vs Uninfected (blue) human AT2 cells. (C-D) Volcano plot of DEGs (C) and GO Biological Process enrichment (D) of genes in Uninfected vs Early IAV (orange) human AT2 cells. (E-F) Volcano plot of DEGs (E) and GO Biological Process enrichment (F) of genes in Early IAV vs Late IAV (red) human AT2 cells. (G) Temporal dynamics of enrichment of GO terms from B, D, and F. Letter to the left of the process name matches letters to the right of the GO term bars. Some terms are enriched in multiple timepoints. Diagrams in G show the dynamics of change of associated terms (boxes) across cell states in AEOs.

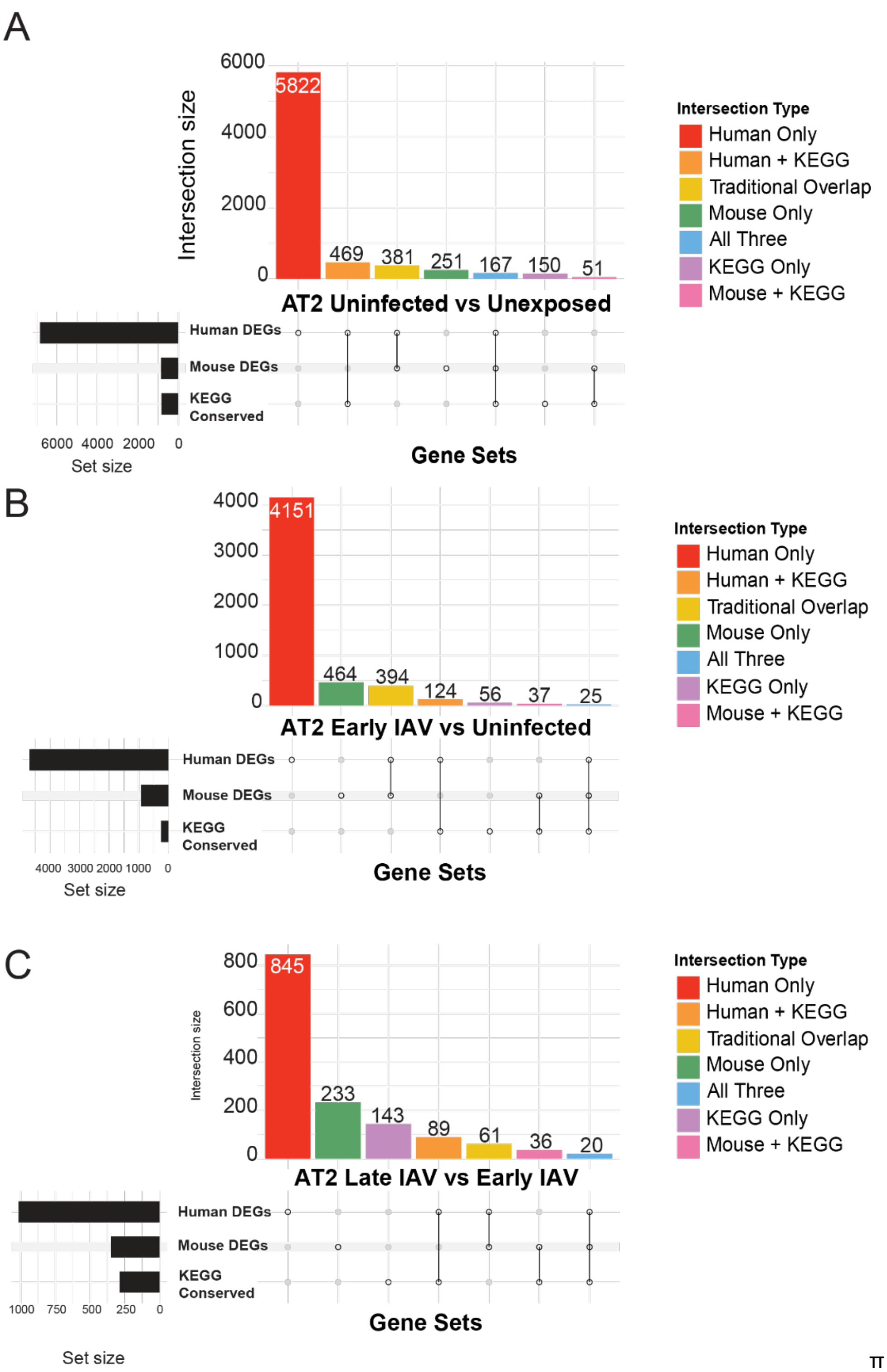

Supplementary Figure 7. Upset plots of functional vs ortholog contribution to the murine and human integration analysis. (A-C) Upset plots of Uninfected vs Unexposed (A), Unexposed vs Early IAV (B), and Early IAV vs Late IAV (C) gene detection for mouse and human conserved IAV response analysis showing the breakdown of shared genes and pathways detected between mouse and human AT2s by comparison.

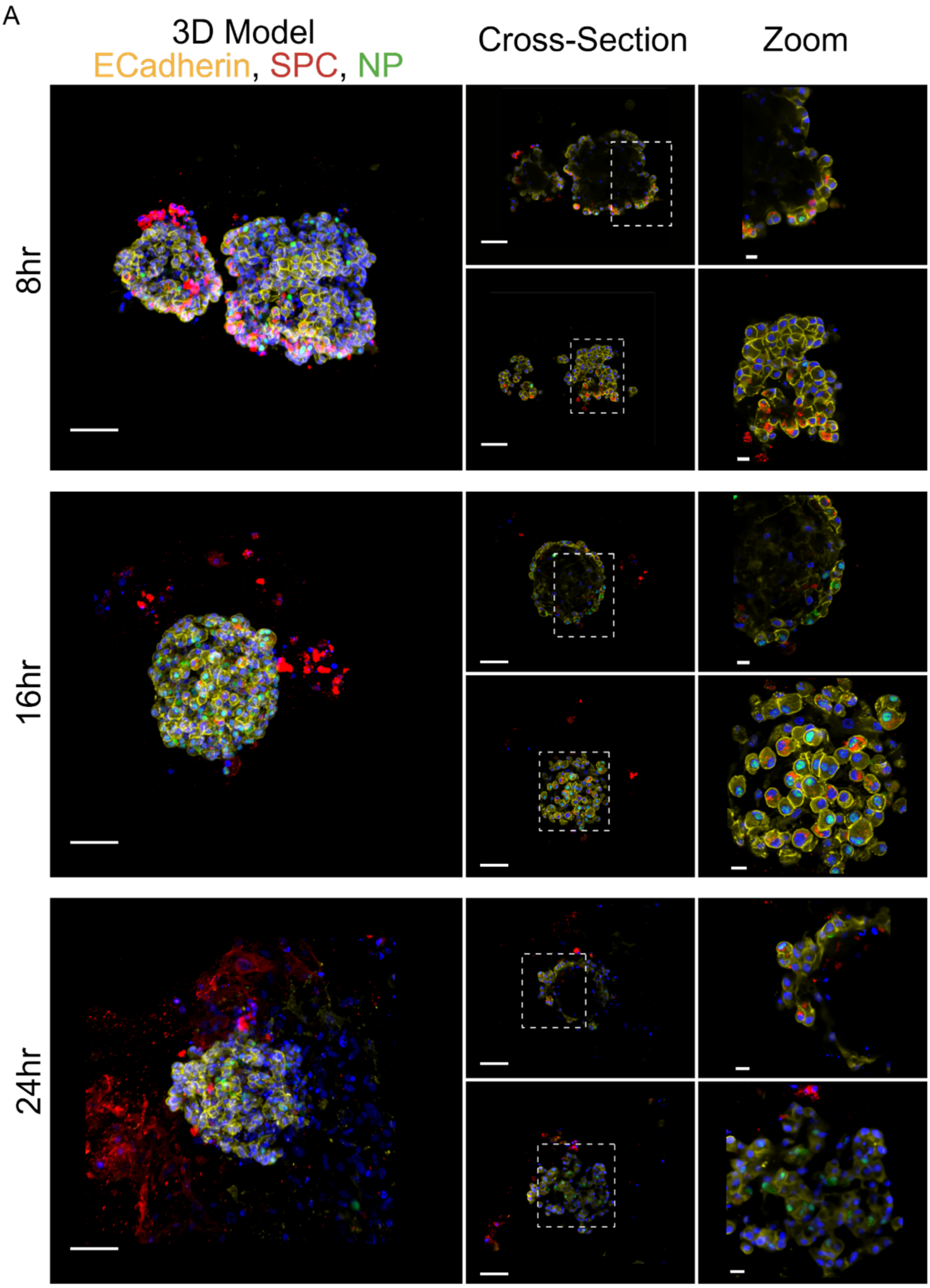

Supplementary Figure 8. SPC loss occurs in infected cells early with increasing loss in uninfected cells later. (A) Murine organoids at 8, 16, and 24hpi with representative cross sections at the top and center of the organoids to shows increasing SPC loss in NP^- cells corresponding to GO-BP findings of lipid dysregulation. Scale bars = 50μm and 10μm for 'Zoom'

Supplemental Table 1: Antibodies used for organoid wholemount immunofluorescence

Primary antibody (1:100)	Secondary antibody (1:200)
**anti-RAGE** (Rat, R&D Systems, MAB1179)	**Donkey anti-Rat IgG Alexa Fluor™ 488** (Invitrogen, A21208)<br><br>**Donkey anti-Rat IgG Alexa Fluor™ Plus 647** (Invitrogen A48272)
**anti-SPC** (Guinea pig, Seven Hills, GP992 and GP993)	**Goat anti-Guinea Pig IgG Alexa Fluor™ 555** (Invitrogen, A21435)
**anti-Influenza NP** (Rabbit, Abcam, ab 308393)	**Donkey anti-Rabbit IgG Alexa Fluor™ 647** (Invitrogen A31573)
**SNA-FITC (**Invitrogen L32479**)**	N/A

Supplemental Table 2: Antibodies used for FACS and spectral flow analysis

Antibody	Supplier	Dilution
Podoplanin-BV421 (8.1.1)	Biolegend, 127423	1:100
CD31-eFluor450 (390)	ThermoFisher (Invitrogen), 48-0311-82	1:100
MHCII-BV480 (M5/114.15.2)	Fisher (BD Horizon), BDB566088	1:300
CD326-BV785 (G8.8)	Biolegend, 118245	1:100
CD24 (M1/69) PE/Cy7	Biolegend, 101821	1:500
CD200-PE/Dazzle594 (OX2)	Biolegend, 123819	1:300
CD326-APC (G8.8)	ThermoFisher (Invitrogen), 17-5791-82	1:100
ItgB4-AlexaFluor647 (346-11A)	Biolegend, 123607	1:100
CD45-AlexaFluor700 (30-F11)	Biolegend, 103127	1:200
Fixable Viability Dye eFluor780	ThermoFisher 65-0865-14	1:1000
CD45 (30-F11) eFluor 450	eBioscience/ThermoFisher 48-0451-82	1:100
SNA-FITC	Invitrogen L32479	1:800

Supplemental File 1: GO Term Comparisons and Leading Edge Genes For Murine and Human AEOs
